# Addressing antimicrobial resistance with the IDentif.AI platform: Rapidly optimizing clinically actionable combination therapy regimens against nontuberculous mycobacteria

**DOI:** 10.7150/thno.73078

**Published:** 2022-09-25

**Authors:** Devika Mukherjee, Peter Wang, Lissa Hooi, Vedant Sandhu, Kui You, Agata Blasiak, Edward Kai-Hua Chow, Dean Ho, Pui Lai Rachel Ee

**Affiliations:** 1 Department of Pharmacy, Faculty of Science, National University of Singapore, Singapore 117545.; 2 The Institute for Digital Medicine (WisDM), Yong Loo Lin School of Medicine, National University of Singapore, Singapore 117456; 3 The N.1 Institute for Health (N.1), National University of Singapore, Singapore 117456; 4 Department of Biomedical Engineering, College of Design and Engineering, National University of Singapore, Singapore 117583; 5 Cancer Science Institute of Singapore, National University of Singapore, Singapore 117599; 6 Department of Pharmacology, Yong Loo Lin School of Medicine, National University of Singapore, Singapore 117600.; 7 NUS Centre for Cancer Research (N2CR), Yong Loo Lin School of Medicine, National University of Singapore, Singapore 117599; 8 Bia-Echo Asia Centre for Reproductive Longevity and Equality (ACRLE), Yong Loo Lin School of Medicine, National University of Singapore, Singapore 117600

**Keywords:** Combination Therapy, *Nontuberculous Mycobacteria*, * Mycobacterium abscessus*, Artificial Intelligence, Infectious Disease

## Abstract

**Background:** Current standard of care (SOC) regimens against nontuberculous mycobacteria (NTM) usually result in unsatisfactory therapeutic responses, primarily due to multi-drug resistance and antibiotic susceptibility-guided therapies. In the midst of rising incidences in NTM infections, strategies to develop NTM-specific treatments have been explored and validated.

**Methods:** To provide an alternative approach to address NTM-specific treatment, IDentif.AI was harnessed to rapidly optimize and design effective combination therapy regimens against* Mycobacterium abscessus* (*M. abscessus*), the highly resistant and rapid growth species of NTM. IDentif.AI interrogated the drug interaction space from a pool of 6 antibiotics, and pinpointed multiple clinically actionable drug combinations. IDentif.AI-pinpointed actionable combinations were experimentally validated and their interactions were assessed using Bliss independence model and diagonal measurement of n-way drug interactions.

**Results:** Notably, IDentfi.AI-designed 3- and 4-drug combinations demonstrated greater %Inhibition efficacy than the SOC regimens. The platform also pinpointed two unique drug interactions (Levofloxacin (LVX)/Rifabutin (RFB) and LVX/Meropenem (MEM)) that may serve as the backbone of potential 3- and 4-drug combinations like LVX/MEM/RFB, which exhibited 58.33±4.99 %Inhibition efficacy against *M. abscessus*. Further analysis of LVX/RFB via Bliss independence model pointed to dose-dependent synergistic interactions in clinically actionable concentrations.

**Conclusions:** IDentif.AI-designed combinations may provide alternative regimen options to current SOC combinations that are often administered with Amikacin, which has been known to induce ototoxicity in patients. Furthermore, IDentif.AI pinpointed 2-drug interactions may also serve as the backbone for the development of other effective 3- and 4-drug combination therapies. The findings in this study suggest that this platform may contribute to NTM-specific drug development.

## Introduction

The incidence of infections caused by nontuberculous mycobacteria (NTM), which are mycobacteria other than *Mycobacterium tuberculosis* (MTB) and *Mycobacterium leprae,* has been increasing at an alarming rate. These organisms are ubiquitous in the environment, but only few species can cause serious and opportunistic infections in immunocompromised patients and those with cystic fibrosis and chronic obstructive pulmonary disease (COPD) 1, 2. The recorded infections and deaths have been rising over the years 3. For example, in the United States from 1997 to 2007, we observed an increase in the annual NTM infections of 8.2% 4. Current protocols to diagnose NTM are complicated with assessments required between clinical, radiological, and microbiological isolation to confirm a diagnosis. Additionally, clinical breakpoints, which are defined for specific pathogens, are inadequate in guiding patient therapy against NTM as it is essentially a diverse group of over 170 species exhibiting varying degrees of antibiotic resistance 1, 5, 6. Even though they are closely related to MTB, NTM usually do not respond to established MTB treatment regimens, as they are intrinsically resistant to many antibiotics. New therapeutic agents are, however, lacking as pharmaceutical companies deprioritize NTM-specific drug development due to the limited market size 7. With insufficient clinical protocols to guide NTM treatment, there is an urgent unmet need for accelerated NTM-specific drug development 8, 9.

Meanwhile, the American Thoracic Society and British Thoracic Society have provided treatment guidelines and dosing recommendations for different species of NTM using existing antibiotics 10-12. The administration of NTM combination therapy regimens typically revolve around drug susceptibility testing and assessment of patient's tolerance to the drugs 13. Current standard of care (SOC) regimens usually result in unsatisfactory treatment outcomes due to the nature of NTM's multi-drug resistance 14. Furthermore, SOC regimens for NTM-related infections are typically prescribed with Amikacin (aminoglycoside antibiotic); however, 39% of patients experienced ototoxicity after 5.5 months (median) of Amikacin in a recent study 12, 15. Though these guidelines provide recommended drug combinations against NTM, further optimization in the design of combination therapies against NTM may lead to the discovery of unforeseen drug interactions and improved clinical outcomes 16, 17. Designing combination therapies and discovering unforeseen drug interactions in clinically actionable concentrations may facilitate the success of the regimens in clinical settings.

Recently, research strategies have been explored to rapidly select effective drug combinations from a pool of candidates, such as synergy prediction and higher-order drug development 18-23. Validations of higher-order drug optimization have demonstrated mechanism-free predictions for multi-drug interactions and provided insight into interactions consisting of three or more drugs 21, 23. However, the clinical relevance of higher-order drug combinations may be challenging as a larger cocktail may achieve similar efficacy than a 2-drug combination, which has the potential to enable higher therapeutic compliance and treatment adherence for patients 24. Even so, simultaneously optimizing drugs and their dosages is still a challenging task in drug development. For example, assessing 10 drugs at 3 dose levels would require rapid screening of over 59,000 combinations. To enable drug discovery and accelerate the development of NTM-specific treatments, we have harnessed an artificial intelligence (AI)-enabled IDentif.AI platform to rapidly optimize and design effective, clinically actionable combination therapy regimens against *Mycobacterium abscessus* (*M. abscessus*)*,* a fast-growing species of NTM that exhibits multi-drug resistance. This platform utilizes a second order quadratic function to describe the correlation between drugs and their corresponding biological response (e.g. %Inhibition). The drug interaction space can then be described by the coefficients arising from the equation and is represented in a smooth response surface. This correlation was first discovered by neural networks and subsequently, validated in multiple *in vitro* studies 16, 25-37 and prospective human studies in infectious diseases, oncology, and many other indications 29, 38-47. This platform does not use synergy predictions, big data on pre-existing drug information, or *in silico* modeling. The IDentif.AI platform only utilizes prospectively obtained *in vitro* percent inhibition (%Inhibition) efficacy against *M. abscessus* to rapidly pinpoint actionable combination therapy regimens as well as non-effective designs that may have no efficacy, making it a versatile, dynamic platform to streamline the workflow for NTM-specific drug development.

**Figure 1:**
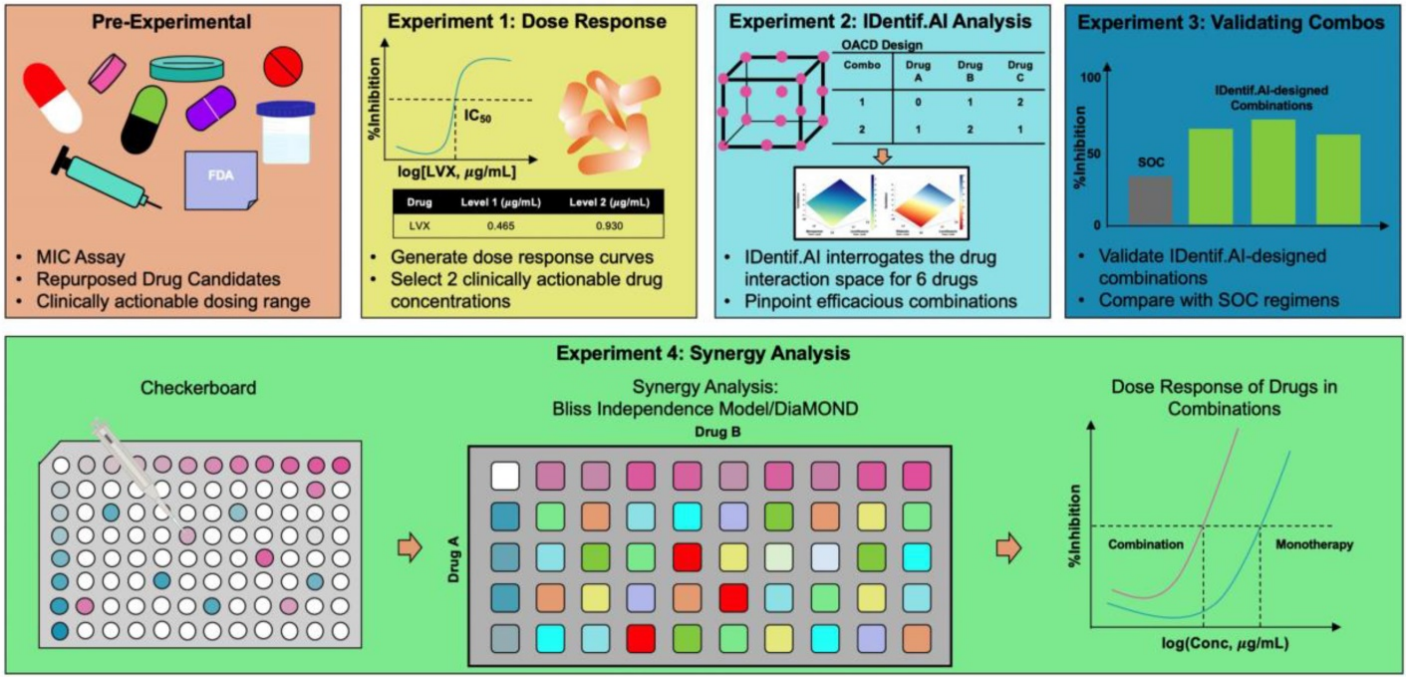
** IDentif.AI workflow to optimize and design combination therapy regimens against *M. abscessus.*** The workflow begins by selecting repurposed drug candidates that are currently used against other NTM species and those that demonstrated *in vitro* efficacy against *M. abscessus* in previous studies. The dose response curves of the selected drugs are then generated, and two clinically actionable concentrations are determined. Subsequently, OACD-designed combinations are experimentally validated, and IDentif.AI harnesses the data to interrogate the drug interaction space. Efficacious IDentif.AI-designed combinations are rapidly pinpointed and subsequently, experimentally validated and compared to standard of care regimens. In the final step, IDentif.AI-pinpointed unique 2-drug combinations are included for further synergy analyses.

In this study, IDentif.AI was harnessed to determine effective combination therapy regimens against NTM from a pool of 6 repurposed drug candidates: Linezolid (LZD), Amikacin (AMK), Meropenem (MEM), Clarithromycin (CLR), Rifabutin (RFB), and Levofloxacin (LVX). The entire IDentif.AI workflow to rapidly optimize and pinpoint drug combinations against *M. abscessus* was completed in 4 steps (Figure 1). IDentif.AI-designed 3- and 4-drug combinations demonstrated promising efficacy against *M. abscessus*. Notably, IDentif.AI-designed LVX/MEM/RFB and LVX/MEM/LZD/RFB combinations exhibited 58.33±4.99 and 55.80±6.56 %Inhibition efficacy against *M. abscessus*, respectively. Additionally, IDentif.AI also pinpointed unique drug interactions in LVX/MEM and LVX/RFB, which displayed greater efficacy than SOC regimens. Subsequent synergy analysis via Bliss independence model and Diagonal Measurement of n-way Drug Interactions (DiaMOND) also revealed mild dose-dependent synergistic interactions within clinically actionable concentrations for the two combinations. LVX/RFB is of special interest as it consists of only orally available drugs, which can potentially increase therapeutic compliance in the prolonged treatment of NTM infections. LVX/MEM and LVX/RFB may also serve as the backbone of other combination therapies and as a useful addition to existing therapies or potentially new therapies which contain these drugs. More importantly, IDentif.AI-designed combinations that do not contain AMK may provide alternative options without AMK-related adverse effects (e.g. ototoxicity). Furthermore, IDentif.AI was able to determine the non-inhibitory effect for RFB/CLR combination against *M. abscessus*, which was subsequently validated *in vitro*. Though no strong synergistic interactions were observed in the interaction space of the 6 repurposed drugs, the IDentif.AI platform has demonstrated its ability to distinguishing optimal and non-effective drug combinations against *M. abscessus*. This suggests that IDentif.AI may potentially be a useful tool in accelerating NTM-specific drug development and to advance current clinical protocols for NTM-related diseases.

## Results

### Initial 6-selected Drug Candidates and *in vitro* Experimental Model

The 6 selected drugs, which have shown efficacy against gram positive and gram negative bacteria and MTB, was selected for preliminary screening against *M. abscessus*. These drugs had different mechanisms of action (MoA) and included protein synthesis inhibitors, DNA/RNA inhibitors, and cell wall synthesis inhibitors. Selecting drugs with varying MoA for combinatorial optimization enables the interrogation of interactions among different drug classes 48. Furthermore, most regimens that are clinically available for NTM-related infections are repurposed from other indications and are not specifically optimized for major pathogens 17. We aimed to specifically optimize and design combination therapies against *M. abscessus* by pairing a wide range of drug classes and repurposed drugs. First, we tested the minimum inhibitory concentrations (MIC) of all 6 drugs against *M. abscessus* to determine their IC_90_, and the selected drugs all showed inhibition against the bacteria. Subsequently, we assessed the clinical actionability of each drug, which included the drug administration route, accessibility, and the potential deployment in a clinical setting. These 6 antibiotics comprised of clinically relevant NTM drugs (AMK and CLR), 2^nd^ line anti-MTB drugs (LZD, MEM, and LVX), and those with *in vitro* activity against *M. abscessus* (LZD and RFB). RFB, which inhibits DNA-dependent RNA polymerase in order to suppress RNA synthesis, has its target in inhibiting *M. abscessus* clinically validated, and it is recommended for use in *M. absecessus* oral treatment 17. AMK, CLR, and LZD inhibit protein biosynthesis, and MEM interferes with the synthesis of cell wall components. LVX inhibits enzymes that are responsible for DNA replication and transcription*.*

The antimicrobial effects were evaluated by exposing *M. abscessus* to drugs for 72 h before measuring the optical density (OD_600_) of treated bacteria and drug free controls in MIC assays to determine the growth inhibition. The efficacy of a drug against *M. abscessus* was measured in percent inhibition (%Inhibition) (Equation S1). To assess the quality of the MIC assays, the spreads of positive (media only; blank) and negative controls (bacteria only; drug free cultures) were used to calculate the Z'-factor (Equation S2). The overall Z'-factor across all experiments was 0.700 (Positive Controls, N = 35; Negative Controls, N = 59), indicating an “excellent” assay 49. Further information on assay quality for each experiment is detailed in the Supplementary Material.

### Dose Response Assessment and Drug Concentration Selection for IDentif.AI Optimization

In the dose response experiment, *M. abscessus* bacteria were exposed to each of the 6 drugs in monotherapy at an increasing concentration (2-fold serial dilutions) (Supplementary Material). The logarithmic scale of drug concentrations was plotted against the respective measured %Inhibitions (N = 2) to construct dose response curves and assess the half-maximal inhibitory concentration (IC_50_), IC_10_, and IC_20_ for each of the 6 antibiotics. The dose response curves for all 6 drugs are illustrated in Figure S1.

To interrogate drug interactions, IDentif.AI assessed two levels (level 1 and level 2) of clinically actionable drug concentrations. The level 1 and level 2 concentrations of each drug were selected based on either IC_10_/IC_20_ or 5%/10% of maximum serum concentration (C_max_). Typically, 10% of C_max_ for a drug is considered as the concentration of drug achievable at the target tissue 16, 37. Thus, the highest level 2 concentrations were selected based on the lower of IC_20_ and 10% of C_max_ to ensure the concentrations evaluated in this study are achievable in the body. The summary of all IC and C_max_ values are shown in Table 1. LZD, MEM, and CLR concentration levels were selected based on IC_10_ and IC_20_, and their level 2 concentrations were restricted within IC_20_ to ensure no overrepresentation of the drugs in IDentif.AI optimization. AMK, RFB, and LVX were selected based on 5% and 10% of C_max_. MIC shifts (2-4*x*) were observed in LVX's IC_20_ in multiple biological replicates and thus, the concentration levels were selected based on C_max_ to ensure clinical actionability. The selected concentration levels are tabulated in Table 2. Additional information pertaining to C_max_ can be found in the Supplementary Material. The ratio between C_max_ and IC_50_ can serve as a measure of a drug's ability to exhibit antimicrobial efficacy when it reaches the maximum concentrations in human blood plasma 16, 50, 51. According to Table 1, of all 6 drugs, only LZD and RFB had low C_max_/IC_50_ ratios (< 1), suggesting that the antimicrobial efficacy may not be sufficient as they reach C_max_.

### Assessing Monotherapies and Optimizing IDentif.AI-designed Combinations

**Figure 2:**
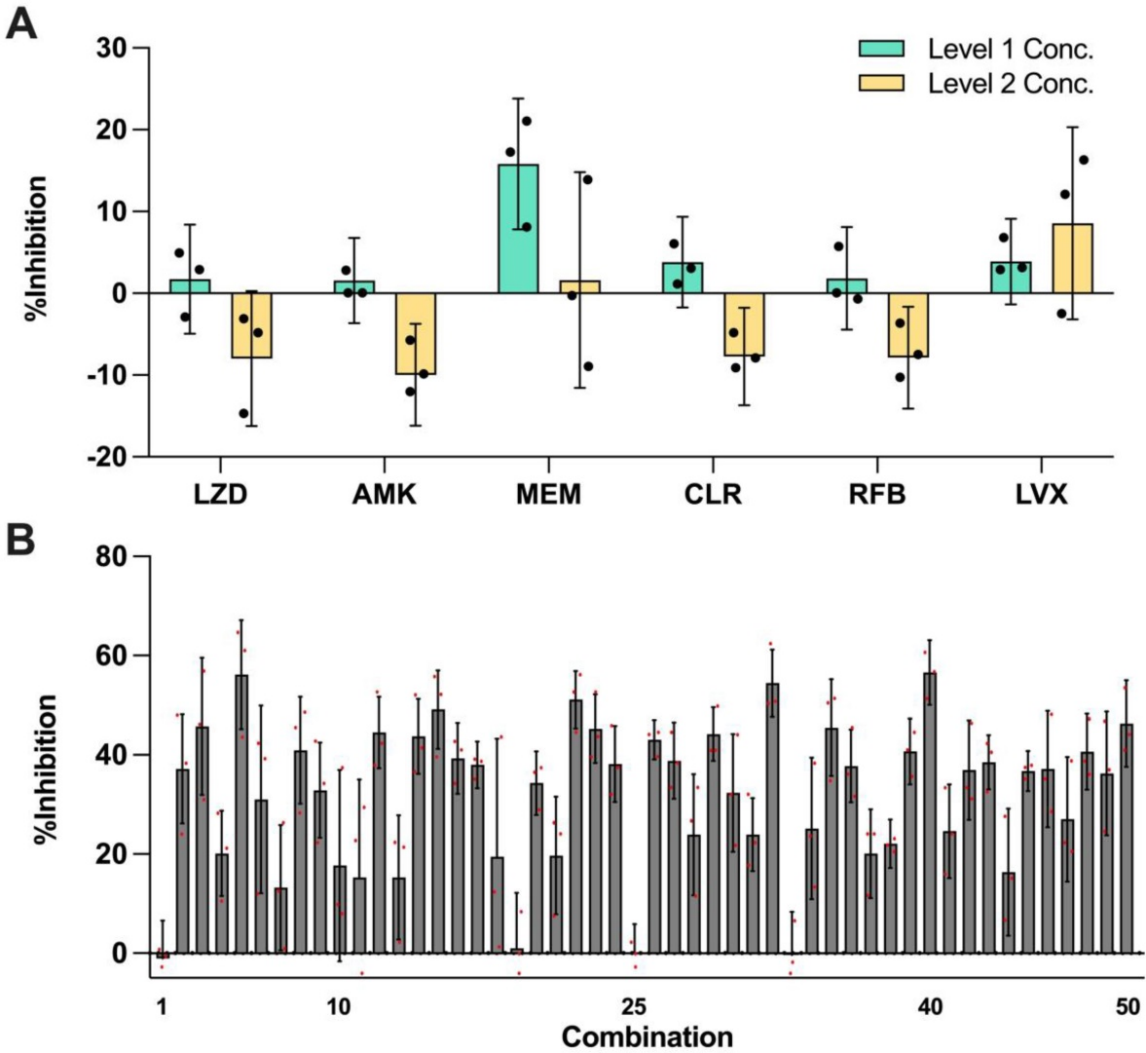
** Efficacy of monotherapies and OACD-designed combinations. (A)** Experimentally measured %Inhibitions for all 6 selected drugs at level 1 (green) and level 2 (yellow) concentrations. The error bars represent the propagated SD, arising from the spread of controls, and each individual replicate is represented in black dots. **(B)** All 50 OACD-designed combinations were experimentally validated, and their corresponding average %Inhibitions are plotted. The combinations are in order in accordance to the design in Table S1, and each replicate is represented in red dots. The monotherapy and combinatorial experiments were performed in the same experiment, and data points are presented as mean ± propagated SD (N = 3). Experimental data are summarized in Table S3 and S4. Level 1 Conc.: level 1 concentration, Level 2 Conc.: level 2 concentration, AMK: amikacin, CLR: clarithromycin, LVX: levofloxacin, LZD: linezolid, MEM: meropenem, and RFB: rifabutin.

In this set of experiment, each of the 6 selected drugs at three concentration levels (level 0 = no drug; level 1 and level 2 represent two clinically actionable concentrations) as specified in Table 2 were experimentally evaluated to determine their monotherapy efficacies against *M. abscessus* (N = 3) (Figure 2A). The %Inhibition efficacies of all 50 OACD-designed combinations were also experimentally obtained via MIC assay (N = 3) (Figure 2B and Table S1).

IDentif.AI analysis harnessed each replicate of the %Inhibition data for OACD-designed combinations (N = 3) and monotherapies for all 6 drugs in level 1 and level 2 concentrations (N = 3), and correlated them using a second order quadratic equation to describe the drug interaction space. Box-Cox transformation did not determine a transformation that improved the distribution of residuals and adjusted R^2^ and therefore, no transformation was applied to the %Inhibition data. No outlier was identified using residual-based outlier analysis, and all replicates (N = 3) were included in the IDentif.AI analysis (Figure S2). The analysis had an adjusted R^2^ of 0.744, indicating a goodness of fit. IDentif.AI-estimated coefficients describing the drug interaction space and statistics are summarized in Table S2. IDentif.AI's interrogation of the drug interaction space pinpointed multiple efficacious drug combinations. The top 3- and 4-drug combinations determined by IDentif.AI with their respective IDentif.AI-predicted %Inhibitions are summarized in Table 3. These combinations were prioritized to facilitate greater potential in clinical deployment, as combinations consisting of 5 or more drugs may result in lower patient adherence, and they may serve as alternative options to SOC regimens that contain AMK, which may induce ototoxicity 15. These IDentif.AI-designed combinations were subsequently validated to assess their efficacies and interactions. All experimental data are summarized in Table S3 and S4.

**Figure 3:**
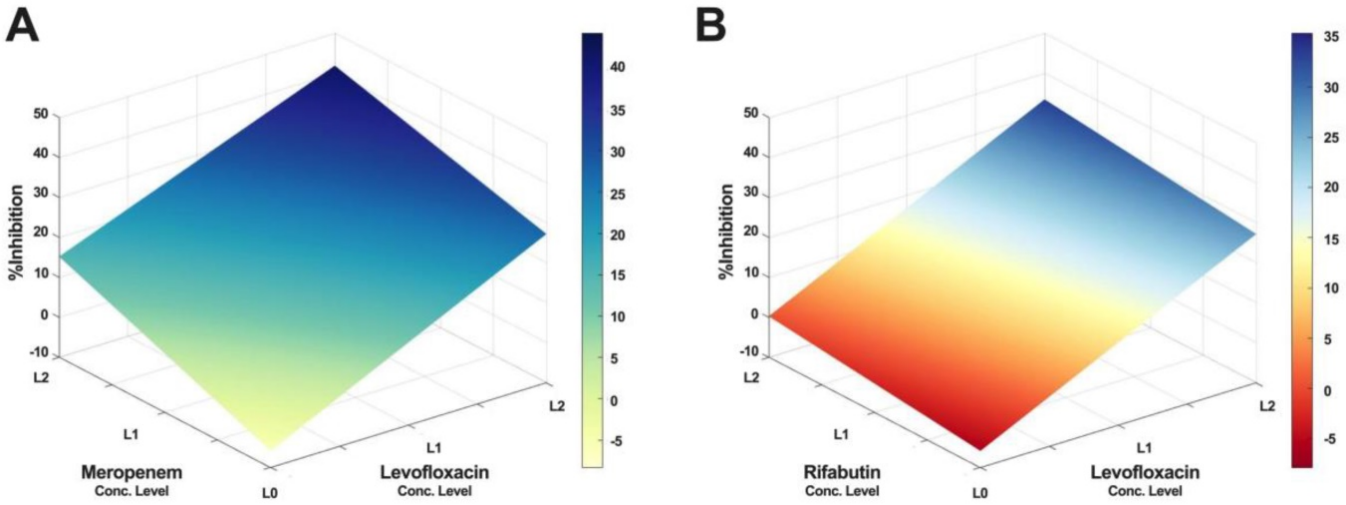
** IDentif.AI drug interaction analysis.** The IDentif.AI analysis identified two 2-drug combinations that may have unique interactions. **(A)** LVX/MEM surface indicated that highest %Inhibition may be achieved when both drugs are at L2, suggesting a synergistic interaction. **(B)** However, LVX/RFB surface suggested that the interaction is mildly synergistic and is mostly driven by LVX. L0, L1, and L2 correspond to the OACD concentration levels: level 0, level 1, and level 2. LVX: levofloxacin, MEM: meropenem, and RFB: rifabutin.

Furthermore, IDentif.AI drug interaction analysis identified unique drug interactions in LVX/MEM and LVX/RFB combinations (Figure 3). LVX/MEM was predicted to achieve highest %Inhibition when both drugs are at level 2, suggesting an interaction between LVX and MEM. Furthermore, IDentif.AI pointed to a mild interaction between LVX and RFB, and determined that the interaction was mostly driven by LVX even with the presence of RFB. Note that LVX/RFB is the only 2-drug combination consisting of orally available drugs. These unique interactions may serve as the backbone of other combinatorial designs (e.g. IDentif.AI-designed 3-drug combination: LVX/MEM/RFB).

### Validating IDentif.AI-designed Combinations and Standard of Care Regimens

To demonstrate the predictions by IDentif.AI are robust and can be experimentally validated, we selected the top ranked IDentif.AI-designed combinations and performed MIC testing to determine their %Inhibitions against *M. abscessus* (N = 3) (Table 3). LVX/MEM and LVX/RFB at level 2 concentrations, representing the IDentif.AI pinpointed unique interactions, were also included in the validation step (N = 3). To assess the efficacy of IDentif.AI-designed combinations against clinically relevant regimens, two SOC regimens were included in the evaluation: AMK/CLR/RFB and AMK/CLR/LZD (N = 3) 10, 11, 52. The IDentif.AI platform can rapidly pinpoint optimal combinations and non-effective combinations that should be avoided in drug development. In this case, three IDentif.AI-pinpointed non-effective combinations that had predicted non-inhibitory efficacy against *M. abscessus* were experimentally validated in this set of experiment (N = 3).

**Figure 4:**
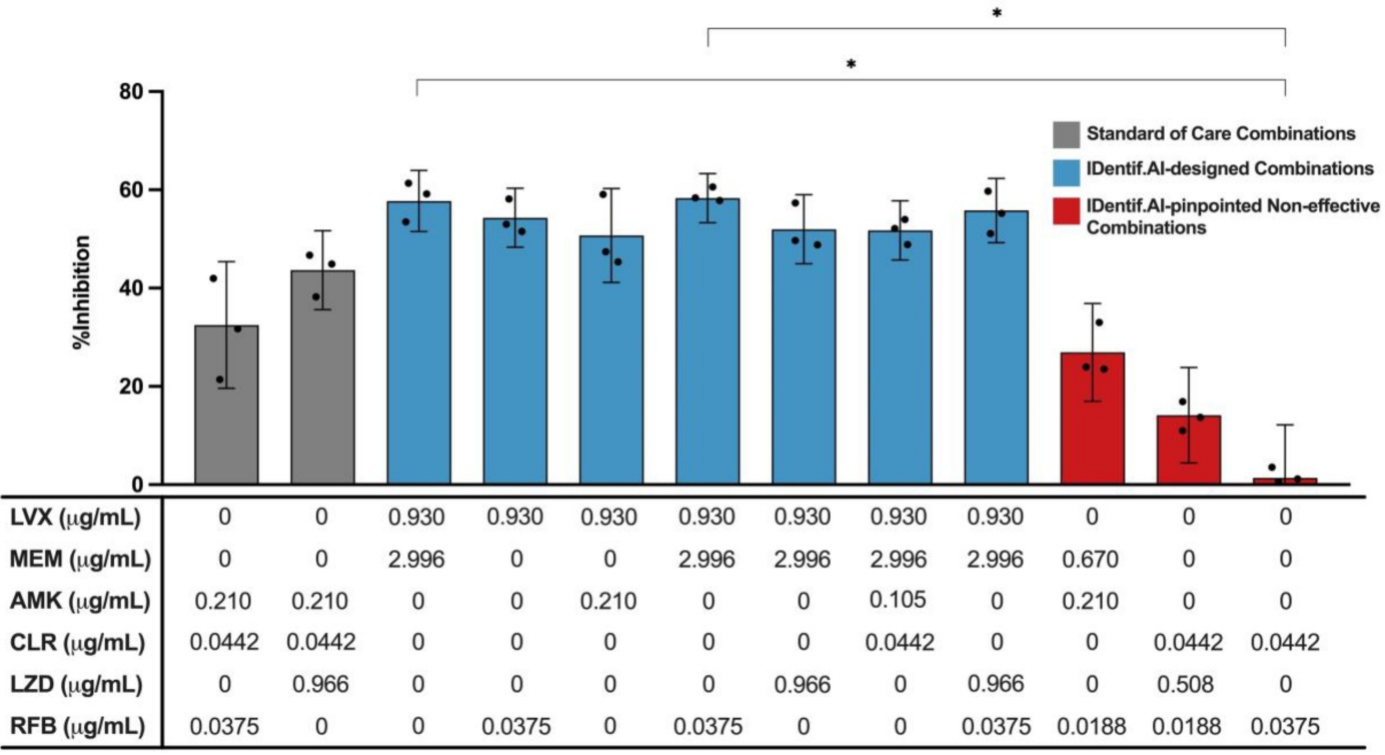
** Validation of standard of care and IDentif.AI-designed combinations against *M. abscessus*.** Two SOC combinations (gray) were validated and compared to IDentif.AI-designed combinations (blue). Furthermore, IDentif.AI-pinpointed non-effective combinations (red) were also experimentally validated. The concentrations of each drug in each combination are listed in the table below. All combinations were experimented in triplicates (N = 3). Each replicate is represented in black dots. Data points are presented as mean ± propagated SD. The error bars represent the propagated SD, which is the measure of the plate-plate variation, instead of the spread of the triplicates. Kruskal-Wallis test detected statistically significant differences at *P* < 0.01 for the %Inhibitions among all validated combinations. Subsequently, pairwise comparisons via Dunn's post hoc test identified statistically significant differences in two pairs of combinations: (1) LVX/MEM and RFB/CLR (2) LVX/MEM/RFB and RFB/CLR (*P < 0.05). Experimental data are summarized in Table S5 and S6. AMK: amikacin, CLR: clarithromycin, LVX: levofloxacin, LZD: linezolid, MEM: meropenem, and RFB: rifabutin.

Figure 4 illustrates the %Inhibitions of SOC combinations (gray), IDentif.AI-designed combinations (blue), and IDentif.AI-pinpointed non-effective combinations (red). IDentif.AI-designed 3- and 4-drug combinations demonstrated greater efficacy than the SOC regimens. Conventionally, NTM and MTB infections are treated with combinations consisting of 3 or 4 drugs as 2-drug combinations may lead to drug resistant strains during the prolonged treatment. However, in this study, IDentif.AI pointed to unique interactions in LVX/MEM and LVX/RFB, which also outperformed the two SOC combinations *in vitro*. Notably, LVX in combination with RFB was the only combination consisting of orally available drugs, and represents a clinically relevant combination that may potentially be implemented into NTM clinical protocols. Importantly, these IDentif.AI pinpointed unique interactions (e.g. LVX/RFB) may serve as the backbone of other combinations and as a useful addition to existing therapies or potentially new therapies which contain these drugs. For example, LVX/RFB served as the backbone of LVX/MEM/RFB and LVX/MEM/LZD/RFB combinations, which exhibited 58.33±4.99 and 55.80±6.56 %Inhibition efficacy, respectively. Additionally, combinations without AMK may serve as alternative regimens for SOC's that contain AMK, which has been known to induce ototoxicity. However, no statistically significant difference was detected between SOC and IDentif.AI-designed combinations via Kruskal-Wallis test and Dunn's post hoc test. All experimental data are summarized in Table S5 and S6.

The non-effective combinations (red) were experimentally validated to have non-inhibitory *in vitro* efficacy against *M. abscessus* (Figure 4). In this experiment, LVX/RFB exhibited 54.32±6.01% in %Inhibition (N = 3), while RFB/CLR demonstrated non-inhibitory *in vitro* efficacy against *M. abscessus* (1.39±10.80%; N = 3) (Figure 4 and S3). The results were in line with the predictions of IDentif.AI, and further indicated that properly administering drugs in combinations is critical to achieve optimal efficacy against *M. abscessus* and other indications. The experimentally measured %Inhibitions for all validated combinations are summarized in Table 3, S5, and S6.

### Synergy Analysis of LVX/RFB and LVX/MEM

**Figure 5:**
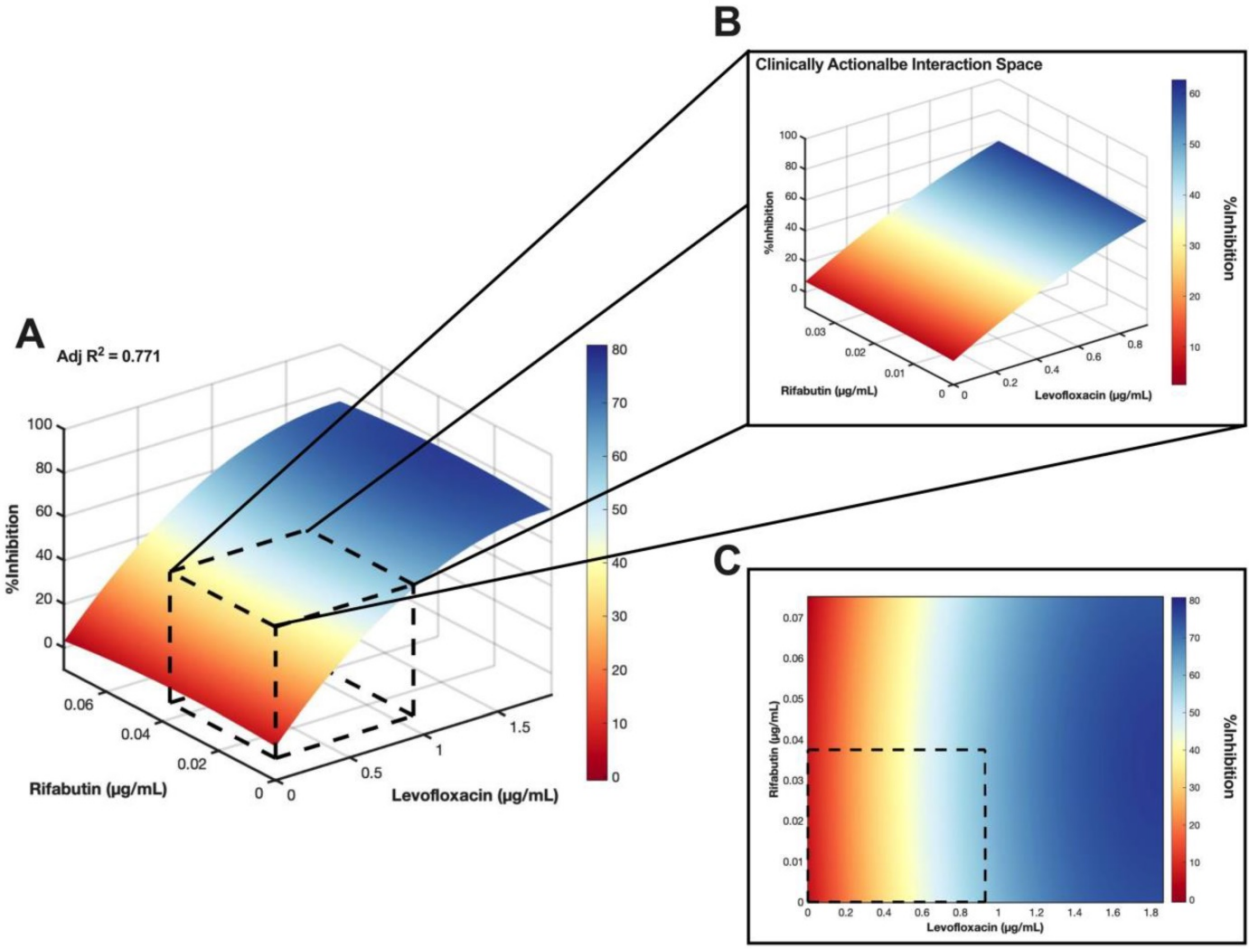
** Validation of LVX/RFB drug interaction space. (A)** Response surface of LVX/RFB in the validation interaction space (0% - 20% C_max_). All replicates (N = 3) were included for the construction of the response surface. The clinically actionable interaction space (< 10% C_max_) is within the dotted black box. The adjusted R^2^ indicates the goodness of the fit for the response surface. **(B)** Clinically actionable interaction space is the magnification of the dotted black box in Figure 5A. **(C)** The heatmap represents the 2-dimensional view of the LVX/RFB response surface in the validation interaction space (0% - 20% C_max_). The clinically actionable interaction space (< 10% C_max_) is within the dotted black box. Experimental data are summarized in Figure S5 and Table S7. Statistics for the response surface are detailed in Table S8. LVX: levofloxacin and RFB: rifabutin.

**Figure 6:**
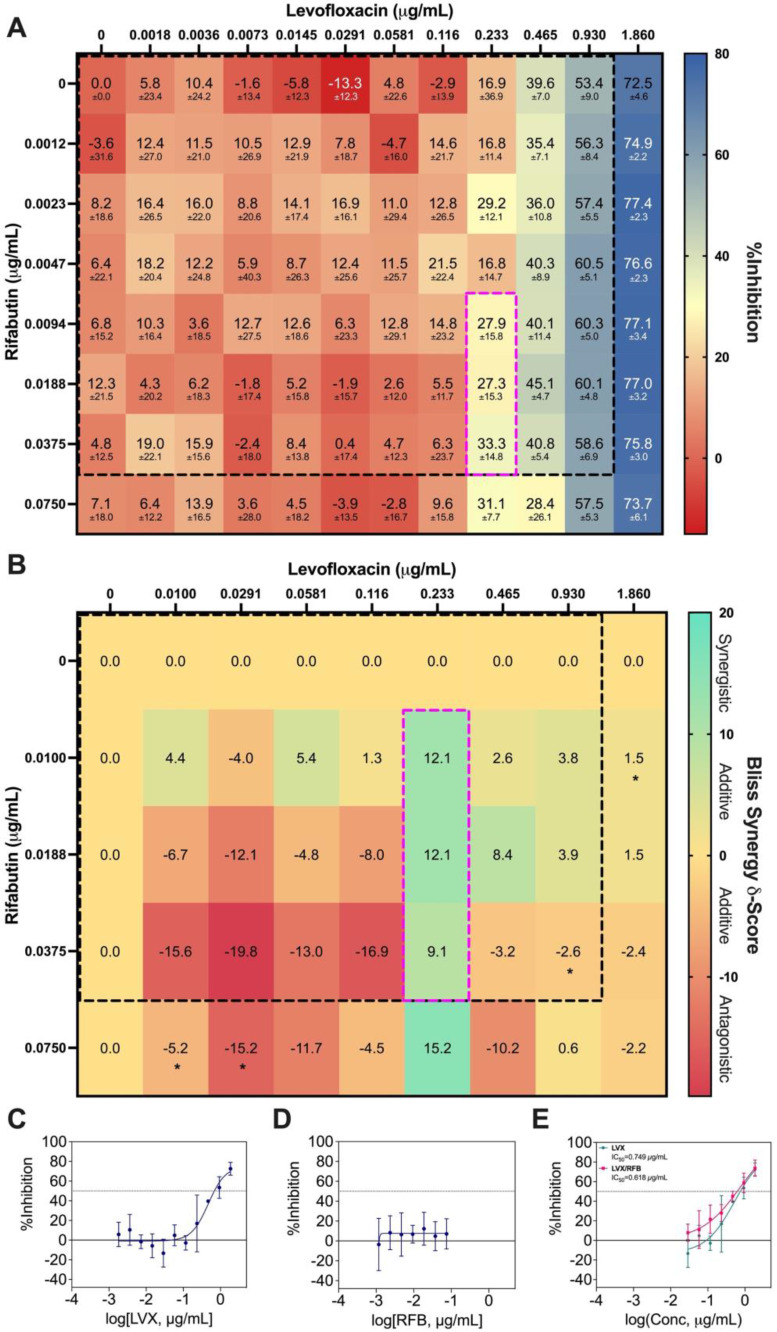
** Bliss independence model analysis of LVX/RFB. (A)** Interaction map of LVX/RFB with measured %Inhibitions at each corresponding dose ratio. The %Inhibitions of LVX/RFB combinations and monotherapies were tested from 0% to 20% C_max_, and the clinically actionable interaction space (< 10% C_max_) for LVX/RFB is within dotted black box, which was also the interaction space analyzed by IDentif.AI (N = 3). **(B)** Synergy map of LVX/RFB with corresponding Bliss Synergy δ-Scores. Scores greater than 10, between -10 and 10, and less than -10 are considered synergistic, additive, and antagonistic, respectively. Bliss independence analysis revealed a mild synergistic dose region (dotted pink box). Clinically actionable interaction space (< 10% C_max_) is within the dotted black box. Statistical significance of the Bliss Synergy δ-Scores was determined by one-sample *t*-test (*P < 0.05). **(C-D)** Dose response curves of LVX and RFB in monotherapies. The dotted line represents absolute IC_50_. Data points are presented as mean ± propagated SD (N = 3). **(E)** Dose response curve of LVX in monotherapy and in combination with RFB. The IC_50_ values for LVX and LVX/RFB are summarized in the legend. The dotted line represents absolute IC_50_. Data points are presented as mean ± propagated SD (N = 3). Experimental data are summarized in Table S7. LVX: levofloxacin and RFB: rifabutin.

As LVX/RFB may potentially be deployable for clinical implementation for the aforementioned reasons, we conducted a checkerboard assay to determine the drug interactions between LVX and RFB. The concentration range tested for LVX was 0.00182 μg/mL to 1.860 μg/mL (2*x* of level 2 concentration; 20% C_max_), and 0.00117 μg/mL to 0.0750 μg/mL (2*x* of level 2 concentration; 20% C_max_) for RFB. The %Inhibitions of the entire checkerboard were obtained via MIC assay (Figure 5A, 6A, and S4A), and all replicates (N = 3) were included to construct the response surfaces (Figure 5). The response surfaces indicated that the %Inhibition was mostly driven by LVX and mildly affected by the presence of RFB (Figure 5 and S4). For instance, LVX in monotherapy (1.860 μg/mL) was able to achieve 72.49±4.57 %Inhibition (N = 3) against *M. abscessus*, and LVX (1.860 μg/mL) in combination with RFB (0.0750 μg/mL) was able to achieve 73.74±6.13 %Inhibition (N = 3). In the clinically actionable interaction space (< 10% C_max_), which was also the interaction space analyzed by IDentif.AI (Figure 5B and S4B), *M. abscessus* demonstrated similar bacterial responses when exposed to LVX/RFB as predicted by IDentif.AI drug interaction analysis (Figure 3B). All experimental data are summarized in Table S7. Statistics for the response surface is detailed in Table S8.

The %Inhibition data were subsequently analyzed using the Bliss independence model 53, 54. The synergy map in Figure 6B displayed the Bliss Synergy δ-Scores, which quantify the strength of synergy based on a given combination and its respective monotherapy efficacy, for the entire dose region (0% - 20% C_max_). Notably, the synergy map identified a region of synergistic dose region (dotted pink box) with Bliss Synergy δ-Score = 12.1, and the drug concentrations are within 10% C_max_. This further suggests that LVX/RFB has mild dose-dependent synergy in the clinically actionable range. In the synergy map, the highest observed Bliss Synergy δ-Score was 15.2, which corresponded to the region when RFB was at 0.0750 μg/mL. However, in this region, RFB was greater than 10% C_max_, indicating that the concentration may not be achievable in human blood plasma. This observed interaction suggests that LVX/RFB may have stronger synergistic interactions beyond the tested 20% C_max_. Figure 6C and 6D illustrate the dose response curves of LVX and RFB, respectively, from this experiment. The dose response curve of RFB revealed that within the tested range, RFB individually had minimal %Inhibition against *M. abscessus*. However, a shift in LVX dose response when administered in combination with RFB was detected (Figure 6E). The IC_10_ and IC_20_ for LVX in monotherapy were 0.167 μg/mL and 0.259 μg/mL, respectively, and the IC_10_ and IC_20_ for RFB were not observed in the tested range. However, in combination with RFB, LVX's IC_10_ and IC_20_ were 0.0438 μg/mL and 0.122 μg/mL, respectively. There is an observed 4-fold reduction in LVX concentration required to achieve 10 %Inhibition of *M. abscessus* when administered in combination with RFB, further suggesting the mild dose-dependent synergistic interaction between the two drugs. Additionally, LVX/RFB may also serve as the backbone of other 3- and 4-drug combinations. All experimental data are summarized in Table S7.

**Figure 7:**
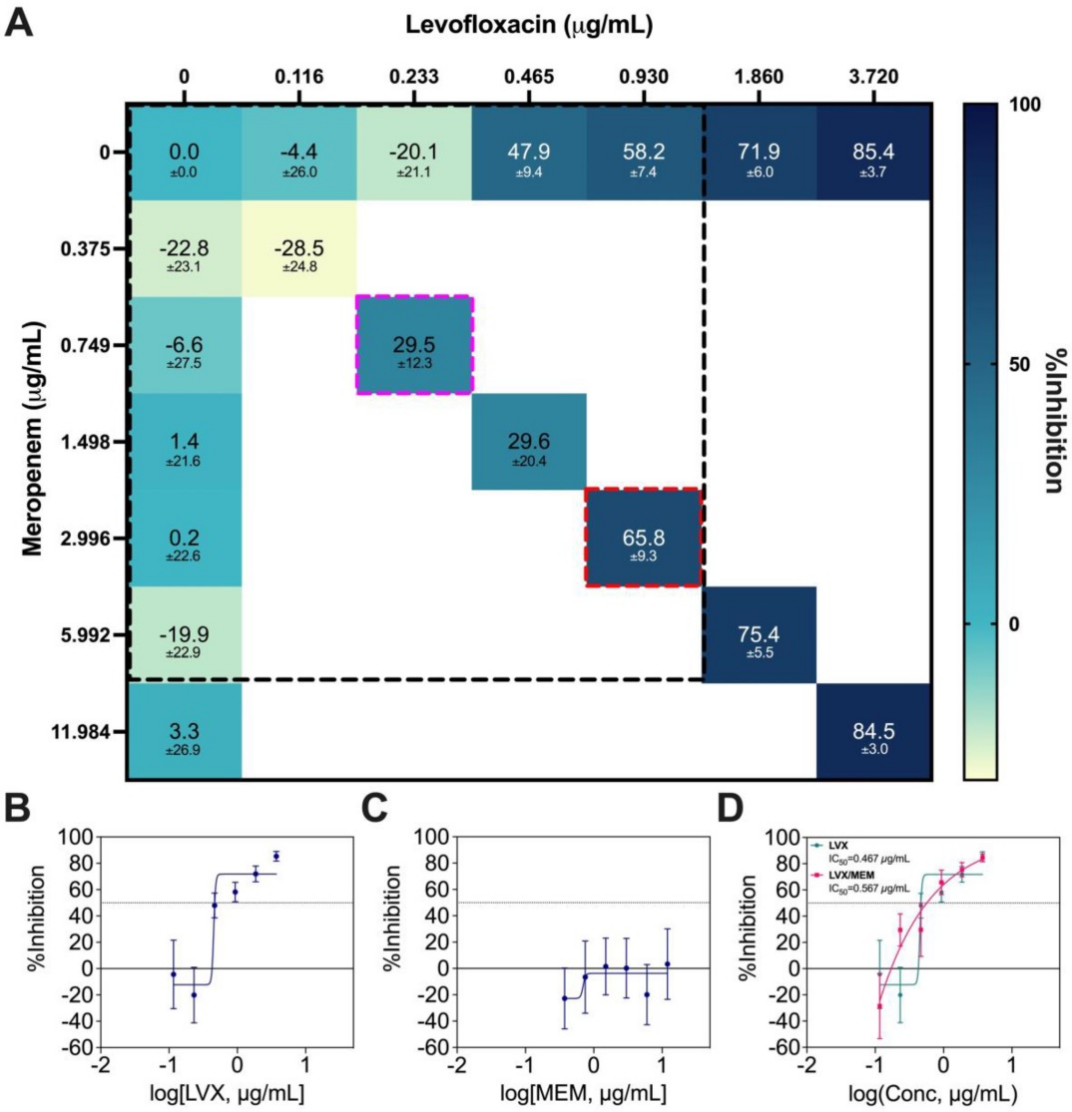
** DiaMOND synergy analysis of LVX/MEM. (A)** Interaction map of LVX/MEM with measured %Inhibitions at each corresponding dose ratios, and the clinically actionable interaction space (< 10% C_max_) is within the dotted black box. The dotted red box represents the combination in original IDentif.AI concentrations (level 2), and the dotted pink box represents the 4-fold MIC shift of the originally identified synergy (N = 3). **(B-C)** Dose response curves of LVX and MEM in monotherapies, and the dotted line represents absolute IC_50_. Data points are presented as mean ± propagated SD (N = 3). **(D)** Dose response curve of LVX in monotherapy and in combination with MEM. The IC_50_ values for LVX and LVX/MEM are summarized in the legend. The dotted line represents absolute IC_50_. Data points are presented as mean ± propagated SD (N = 3). Experimental data are summarized in Table S9. LVX: levofloxacin and MEM: meropenem.

LVX/MEM was the optimal 2-drug combination pinpointed by IDentif.AI, but MEM is not an orally available drug. As a result, this combination was not prioritized in terms of ease of deployment and therapeutic compliance. Therefore, only a Diagonal Measurement of n-way Drug Interactions (DiaMOND) synergy analysis, which is the geometric optimization of the checkerboard assay, was performed to assess the drug interactions (Figure 7A) 55. The concentration range tested was 0.375 μg/mL to 3.720 μg/mL for LVX, and 0.116 μg/mL to 11.984 μg/mL for MEM. The same ratio of the original IDentif.AI-pinpointed LVX/MEM concentrations was retained (dotted red box), and synergy was observed in LVX/MEM (dotted pink box) at exactly 4-fold reduction from the original ratio (dotted red box) (Figure 7A). The data from DiaMOND synergy analysis were used to construct dose response curves for LVX and MEM in monotherapies (Figure 7B and 7C). MEM within the tested concentration range had no dose response against *M. abscessus*. A shift in LVX's dose response, especially in lower concentrations, is illustrated in Figure 7D. LVX in monotherapy can only achieve IC_10_ and IC_20_ at 0.437 μg/mL and 0.445 μg/mL, respectively. However, LVX in combination with MEM can achieve IC_10_ and IC_20_ at 0.205 μg/mL and 0.252 μg/mL, respectively. At lower concentrations, LVX/MEM can achieve greater %Inhibition, while LVX individually had non-inhibitory effect against *M. abscessus*. This finding is in line with the mild dose-dependent synergy demonstrated in Figure 7A. Similar to LVX/RFB, this combination may also serve as the backbone of other 3- and 4-drug combinations. All experimental data are summarized in Table S9.

## Discussion

### Clinical Actionability of IDentif.AI-designed Combinations

IDentif.AI was able to pinpoint 3- and 4-drug combinations in clinically actionable concentrations as summarized in Table 3, and it was also able to detect unique drug interactions (e.g. LVX/RFB). In this study, 2-drug combinations were of special interest as treatment regimens consisting of fewer drugs may potentially increase compliance for infected NTM patients during the prolonged 6-12-month treatment 24, 56. However, most current SOC regimens against NTM consist of 3 or more drugs. Thus, a 2-drug combination that can achieve similar, if not better, efficacy than multi-drug combinations may be favored in terms of therapeutic compliance 24. The potential development of drug resistance as a result of administering 2-drug combination in a prolonged treatment like NTM should also be carefully considered. Combinations consisting of orally available drugs may also lead to greater treatment compliance. To address compliance for regimens that require intravenous (IV) infusion, Outpatient Parenteral Antimicrobial Therapy (OPAT), which enables the administration of IV antibiotics at home or at an outpatient setting, may be implemented 57. In this study, LVX/RFB demonstrated promising efficacy against *M. abscessus,* and its interaction was comprehensively analyzed using Bliss independence model (Figure 6B). We subsequently confirmed mild dose-dependent synergy of LVX/RFB in clinically actionable concentrations. LVX and RFB are two readily available oral drugs in the clinic and thus, LVX/RFB may be conveniently implemented into NTM clinical protocols as a combination or backbone of combination therapies by pairing additional drugs. LVX/MEM was another interesting drug interaction pinpointed by IDentif.AI, but MEM is not orally available. However, LVX/MEM may also serve as a promising backbone of combination therapy regimens against *M. abscessus* if OPAT is readily available to patients. LVX/MEM and LVX/RFB may be prioritized for ease of deployment and greater therapeutic compliance, and they may also serve as the backbones of other potential combination therapies.

To enable a successful treatment strategy specific to NTM infections, the drug resistance that may develop as a result of the administration of 2-drug combinations for the prolonged treatment should be carefully considered. Conventionally, NTM and MTB are treated with combinations consisting of 3 to 4 drugs 11, 12, 58. Measures to reduce the occurrence of drug-resistant strains must also be addressed even though treatment compliance is critical to clinical outcomes. More importantly, IDentif.AI-designed combinations all contained LVX, a fluoroquinolone antibiotic that is highly prone to induce drug resistance 59-61. IDentif.AI pinpointed interactions (LVX/MEM and LVX/RFB) both contained LVX and only exhibited mild interactions, which may lead to drug resistance. They may however serve as the backbone of other combinations. For example, pairing additional drugs to these 2-drug combinations (e.g. IDentif.AI-designed 3-drug combination: LVX/MEM/RFB) may further increase %Inhibition efficacy and reduce the potential of developing drug-resistant strains. IDentif.AI-designed 3- and 4-drug combinations may also have a greater potential in translating into current NTM clinical protocols as established regimens usually contain 3 or 4 drugs.

Furthermore, IDentif.AI is able to optimize and prioritize regimens according to clinical needs and many other factors, such as cost, drug resistance, and adverse effects. IDentif.AI can rapidly determine an optimal combination therapy regimen that includes or excludes a certain drug per clinical indication of a patient. For example, studies have determined that 25-39% of patients administered with AMK experienced ototoxicity. LVX/MEM/RFB and LVX/MEM/LZD/RFB, which do not contain AMK, may provide similar clinical outcome without potential AMK-related adverse effects 15, 62. Similarly, in a region where antibiotic shortages may occur, IDentif.AI then is able to prioritize optimized regimens that only contain readily available drugs. Table 3 also summarizes a list of actionable IDentif.AI-designed combinations against *M. abscessus.* It is important to note that the IDentif.AI workflow summarized in Figure 1 can be conveniently applied to design and optimize combination therapy regimens against other infectious diseases.

### Ongoing Trials and Existing Information on IDentif.AI-designed Combinations

Though no existing clinical trials have reported the use of LVX/MEM for NTM infections, other *in vitro* and *in vivo* studies have identified LVX/MEM as a synergistic regimen against other disease indications. In a study published by Louie *et al.,* LVX/MEM was able to induce 2- to 3-fold reduction in resistance suppression and bacterial kill rate in *Pseudomonas aeruginosa* (PAO1 strain) 63. Following the *in vitro* study, Louie *et al.* conducted a separate *in vivo* validation of LVX/MEM in a murine *Pseudomonas aeruginosa* model. That study found that LVX/MEM exhibited synergy and induced promising bacterial kill rate and resistance suppression against *Pseudomonas aeruginosa* 64. The team suggested a further clinical investigation on LVX/MEM. Moreover, multiple studies have also suggested the clinical actionability of MEM in addressing antimicrobial resistance of ESKAPE (Enterococcus faecium, Staphylococcus aureus, Klebsiella pneumoniae, Acinetobacter baumannii, Pseudomonas aeruginosa, and Enterobacter spp) pathogens 65-68. Together with the IDentif.AI results pointing to a mild dose-dependent interaction between LVX and MEM, these findings stress the need to tailor combinatorial treatment to each pathogen individually.

RFB has been widely recognized as a repurposed drug candidate against *M. abscessus* 69*.* No existing clinical trials or literatures have referenced the administration of LVX and RFB in combination. However, in a trial (NCT04310930), RFB among other repurposed drug candidates serves as interventional drugs to determine the optimal regimens against *M. abscessus*. Multiple ongoing clinical trials are recruiting patients to determine the efficacy of RFB against other species of NTM (NCT03164291; NCT00810407). Though LVX/RFB and LVX/MEM only exhibited mild dose-dependent synergy in clinically actionable concentration ranges *in vitro*, these IDentif.AI-designed combinations did demonstrate >50 %Inhibition efficacy against *M. abscessus* and are worth further investigations on their potential as a combination and as the backbone of combinatorial designs in preclinical and clinical models.

### Rapid Screening of Efficacious Combinations

This study presented an unconventional approach to design combination therapy regimens. Typically, drugs in monotherapies are carefully examined to determine their potency against a given indication (e.g. NTM, SARS-CoV-2). Potent drugs that can achieve IC_50_ at low concentrations are strongly considered in combinatorial designs. However, those that are not potent are often disregarded, similar to susceptibility-guided treatments. In this study, even drugs that require high concentrations to achieve IC_50_ were included in the interrogation of drug interactions. Notably, MEM had relatively low potency against *M. abscessus**,*** and IDentif.AI thereafter pinpointed LVX in combination with MEM as the optimal 2-drug combination. Subsequent synergy analysis demonstrated that MEM in combination with LVX had mild dose-dependent synergy (Figure 7). In Figure 6, RFB monotherapy demonstrated non-inhibitory and minimal efficacy against *M. abscessus.* However, the synergy map pointed to mild dose-dependent synergistic interaction between LVX and RFB (Figure 6B). Thus, drugs that lack potency or efficacy against a given indication in monotherapy may still have interactions when properly administered in combinations.

Novel drug interactions were pinpointed by IDentif.AI using experimental data. In the IDentif.AI analysis, IDentif.AI correlated the relationship between 50 OACD-designed combinations and their respective %Inhibitions to interrogate the interactions of all (3^6^) 729 possible combinations for 6 drugs at three concentration levels and subsequently, provided a ranked list of efficacious and non-effective combinations (Figure 4 and S3 and Table 3). This is the highlight of IDentif.AI, and it is especially critical in the presence of rising incidence of NTM infections and other infectious diseases. Should a new species of NTM emerge with resistance to current SOC's, IDentif.AI may be harnessed to determine effective regimens in a short, actionable period of time.

### IDentif.AI Pinpointed Non-effective Combinations

In this study, IDentif.AI demonstrated its actionability in pinpointing efficacious and non-effective combination therapy regimens against *M. abscessus*. Notably, IDentif.AI determined that RFB in combination with LVX may be one of the optimal combination therapy regimens, and that RFB/CLR may yield non-effective results (Table S6)*.* Subsequent experimental validation confirmed the finding. Figure 4 and S3 show that the three IDentif.AI-pinpointed non-effective combinations had non-inhibitory %Inhibitions in comparison to SOC and IDentif.AI-designed combinations. However, adjusting the dose ratios of these combinations may potentially result in improved efficacy. IDentif.AI therefore may potentially be positioned as a clinical decision support system (CDSS) to properly design effective combinatorial designs to address antimicrobial resistant bacterial infections.

### Limitations of the Study

IDentif.AI-designed combinations were optimized and pinpointed based on *in vitro* experiments. Future evaluations in preclinical models and potentially human trials will require further dose optimization and validation in the respective experimental models. In this study, toxicity profiles of IDentif.AI-designed combinations were not determined. Though the toxicity profiles for all 6 repurposed drugs have been well understood for other indications, some drugs may however induce dose-dependent synergy in toxicity when administered in combinations. Thus, safety and tolerance profiles in subsequent preclinical and clinical models will need to be assessed.

Although it can rapidly optimize combination therapy designs, IDentif.AI has some limitations that require further development to comprehensively pinpoint drug combinations. This platform correlates efficacy (e.g. %Inhibition) to drugs and their respective concentration levels via a second order quadratic relationship. As a result, IDentif.AI's drug interaction analysis and synergy analysis (e.g. checkerboard) were mostly limited to 2-drug combinations. Furthermore, the design of OACD used in this study is only consisted of three concentration/dose levels, which may limit the discovery of synergy in some drugs. For example, LVX/MEM and LVX/RFB exhibited dose-dependent synergistic interactions at different concentration ratios than the IDentif.AI analysis (Figure 6 and 7). Therefore, an OACD design that can incorporate more concentration levels while maintaining a manageable experimental size may enable a better discovery of unforeseen drug interactions within clinically actionable range.

In this study, the AMK/CLR/LZD combination, which was the top performing SOC regimen, and LVX/MEM were able to achieve 43.65±8.04 and 57.69±6.21 %Inhibitions, respectively, and no statistically significant difference was detected when compared to IDentif.AI-designed combinations. Even so, IDentif.AI-designed combinations still represent the globally optimal combinations from the initial pool of 6 drugs. Optimizing a new set of drug candidates may eventually lead to drug combinations that are significantly better than SOC regimens. Continuously optimizing drug combinations from different pool of clinically relevant drugs may facilitate the discovery and development of NTM-specific treatments.

## Conclusions

In summary, this study demonstrated the ability of IDentif.AI to rapidly design and optimize combination therapy regimens against *M. abscessus*. We harnessed IDentif.AI to interrogate the drug interaction space for 6 drugs in clinically actionable concentrations, and the platform rapidly pinpointed a ranked list of actionable drug combinations*.* IDentif.AI-designed 3- and 4-drug combinations demonstrated greater %Inhibitions than the SOC regimens. Furthermore, IDentif.AI pointed to unique interactions in LVX/RFB and LVX/MEM combinations, which exhibited mild synergistic interactions at only selected dose regions. However, IDentif.AI-designed 2-drug combinations may also serve as the backbone of other combination therapies, in order to accelerate the translation of new combination therapies into current NTM clinical protocols. Additionally, IDentfi.AI-designed combinations that do not contain AMK may serve as alternative regimens to the SOC combinations by achieving comparable efficacy against *M abscessus* and reducing the potential of AMK-induced toxicity. The actionability of IDentif.AI in pinpointing both efficacious and non-effective combinations against *M. abscessus* further suggests that this platform may be implemented to streamline NTM-specific drug development.

## Materials & methods

### Bacterial Growth Inhibition Assay

All experiments were conducted in a BSL 2 laboratory. Serial dilutions of the drugs were prepared in a microtiter plate. *M. abscessus subsp abscessus* was cultured in Middlebrook 7H9 broth (BD) supplemented with ADC (Sigma Aldrich). Cultures were grown in mid-log phase and then diluted to a final density of 10^6^ CFU/mL in the drug containing microtiter plates. The plates were incubated for 72 h at 37^o^C with shaking at 120 rpm. After incubation, the cultures were resuspended to give a homogenous distribution and then OD_600_ was measured (Tecan Infinite M2 Plate Reader) as an indicator of bacterial growth. Drug free cultures were used as negative controls. *M. abscessus* growth inhibition was then calculated based on Equation 1. The dose response curves were constructed using GraphPad Prism 9 (GraphPad Software), and the IC_10_, IC_20_, and IC_50_ values were calculated from the curves.

### IDentif.AI Interrogates Drug Interaction Space

The %Inhibitions of all 50 OACD-designed combinations (Table S1) and monotherapies in level 1 and level 2 concentrations (Table 2) were experimentally measured (N = 3). Subsequently, IDentif.AI harnessed the experimentally obtained *in vitro* data for all 50 OACD-designed combinations and monotherapies (N = 3) 70, and correlated the data into a second order quadratic series. Using this equation, the linear, bilinear (drug-drug interaction), and quadratic parameters of each drug can be determined (Table S2). The IDentif.AI correlation follows the quadratic model in Equation 1:



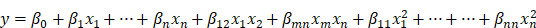



In Equation 1, 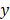 represents the biological response of *M. abscessus* (%Inhibition) with respect to the therapeutics (antibiotics), 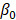 is the intercept term for the quadratic equation, 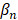 is the coefficient for the *n*th antibiotic, 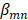 is the interaction terms between *m*th and *n*th antibiotics, and 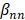 is the second order quadratic coefficient for the *n*th antibiotic. 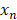 serves as the input of the concentration level (0, 1, or 2) of a given antibiotic. IDentif.AI then performed a stepwise regression to interrogate drug interactions and pinpoint optimal drug combinations (MATLAB R2020b; MathWorks, Inc.). Box-Cox transformation was explored to determine appropriate data transformation on the %Inhibition data to improve residual distributions and the fit of IDentif.AI quadratic model represented by the adjusted R^2^. To assess the %Inhibition data, residual-based outlier analysis was performed. IDentif.AI then utilized the quadratic series to generate a ranked list of all possible combinations for 6 drugs in three concentration levels (3^6^ = 729) and their corresponding IDentif.AI-predicted %Inhibitions. Additionally, IDentif.AI analysis pointed to unique, unforeseen drug interactions via response surfaces that correlated drug concentration levels to %Inhibitions (MATLAB R2020b; MathWorks, Inc.). Example IDentif.AI code written in MATLAB is provided in the Supplementary Material.

### Validating SOC and IDentif.AI-designed Combinations

To validate the efficacy of IDentif.AI-designed combinations against *M. abscessus* in clinically actionable concentrations, selected 2-, 3-, and 4-drug combinations (Table 3) were experimented *in vitro* (N = 3). Two SOC regimens (N = 3) consisted of only drugs from the initial pool of 6 drug candidates were also included in this validation experiment. The efficacies of IDentif.AI-designed combinations were then benchmarked against the SOC regimens to determine if significant improvements in efficacy can be achieved.

### Bliss Independence Model and DiaMOND for Synergy Analysis

LVX/RFB, was experimentally tested in a checkerboard assay (N = 3). LVX concentrations started at 1.86 μg/mL (20% C_max_; 2*x* of level 2 concentration) and serial diluted by a factor of 2 to 0.00182 μg/mL. RFB concentrations started at 0.0750 μg/mL (20% C_max_; 2*x* of level 2 concentration) and serial diluted by a factor of 2 to 0.00117 μg/mL. The drugs were incubated with bacterial cultures for 72 h after which OD_600_ was measured to determine the %Inhibition of the drugs both individually and in combinations. Subsequently, the drug concentrations and corresponding %Inhibition were inputted to GraphPad Prism 9 (GraphPad Software) to generate an interaction map for the entire checkerboard. The data set was subsequently uploaded to SynergyFinder to perform Bliss independence model analysis 53, 54, 71. Synergy scores for each combination were downloaded to generate a synergy map (GraphPad Prism 9; GraphPad Software) (MATLAB 2020b; MathWorks, Inc.).

The interaction of LVX/MEM was assessed via a DiaMOND synergy analysis (N = 3) with similar incubation conditions as the checkerboard assay 55. The concentrations for LVX and MEM were at 3.720 μg/mL and 11.984 μg/mL (4*x* the original level 2 concentrations), respectively, and they were serial diluted to 0.116 μg/mL and 0.375 μg/mL, respectively. The interaction map was generated using GraphPad Prism 9 (GraphPad Software).

### Statistical Analysis

All *in vitro* experiments were performed in 3 biological replicates, except for the dose response experiment, which was performed in 2 biological replicates. Experimentally derived data are presented as mean ± propagated SD (Equation S1 and S3) 72. The IDentif.AI analysis and its estimated coefficients were analyzed using sum of square *F*-test. The *P*-values of IDentif.AI-estimated coefficients served as coefficient exclusion criteria for stepwise regression (Table S2) 16, 37. The distribution of the %Inhibition data for experimentally validated combinations was tested using the Shapiro-Wilk normality test 16, 37. For multiple comparison and pairwise comparison, Kruskal-Wallis test and Dunn's post hoc test were performed, respectively 16, 37, 73, 74. The statistical significance of synergy scores obtained via Bliss independence model was determined using one-sample *t*-test.

## Figures and Tables

**Table 1:** ** Efficacy of the 6 selected dugs against *M. abscessus*.** Absolute IC_10_, IC_20_, and IC_50_ were obtained from dose response curves of each drug individually based on MIC assay with *M. abscessus*. The C_max_ values were obtained from FDA regulatory documents and literatures as outlined in the Supplementary Material. The NationalClinicalTrial.gov (NCT) identifiers of trials pertaining to the administration of the corresponding drugs against NTM infections are also listed. AMK: amikacin, CLR: clarithromycin, LVX: levofloxacin, LZD: linezolid, MEM: meropenem, and RFB: rifabutin.

Drug	(μg /mL)			(μg /mL)			
	IC_10_	IC_20_	IC_50_	C_max_	5% C_max_	10% C_max_	Clinical Trial
LZD	0.508	0.966	6.567	12.7	0.635	1.270	NCT03220074
AMK	0.917	1.490	4.413	2.1	0.105	0.210	NCT01315236
MEM	0.670	2.996	22.280	61.6	3.080	6.160	/
CLR	0.0325	0.0442	0.129	10	0.500	1.000	NCT00600769
RFB	3.628	4.412	6.756	0.375	0.0188	0.0375	NCT00810407
LVX	0.656	0.663	0.680	9.3	0.465	0.930	NCT03220074

**Table 2:** ** Clinically actionable drug concentration levels for IDentif.AI's interrogation of drug interactions.** Concentration levels corresponding to the OACD design are level 0, which is the absence of a drug, and level 1 and level 2 representing two clinically actionable drug concentrations 70. Level 1 and level 2 concentrations were selected based on absolute IC_10_ and IC_20_ for LZD, MEM, and CLR. Concentrations for AMK, RFB, and LVX were selected based on 5% and 10% C_max_. AMK: amikacin, CLR: clarithromycin, LVX: levofloxacin, LZD: linezolid, MEM: meropenem, and RFB: rifabutin.

Drug	Level 0 (μg /mL)	Level 1 (μg /mL)	Level 2 (μg /mL)
LZD	0	0.508	0.966
AMK	0	0.105	0.210
MEM	0	0.670	2.996
CLR	0	0.0325	0.0442
RFB	0	0.0188	0.0375
LVX	0	0.465	0.930

**Table 3:** IDentif.AI-designed combinations and their corresponding, rank, concentrations, and %Inhibitions are listed below. The rank is out of 729 total possible combinations. Data points are presented as mean ± propagated SD (N = 3).

Rank	Top 2-Drug Combinations				IDentfi.AI %Inhibition	Measured %Inhibition
190	Levofloxacin (0.930 μg/mL)	Meropenem (2.996 μg/mL)			41.84	57.69±6.21
339	Levofloxacin (0.930 μg/mL)	Meropenem (0.670 μg/mL)			34.54	50.40±5.38
341	Levofloxacin (0.930 μg/mL)	Amikacin (0.105 μg/mL)			34.41	/
363	Levofloxacin (0.930 μg/mL)	Rifabutin (0.0375 μg/mL)			33.41	54.32±6.01
396	Levofloxacin (0.930 μg/mL)	Amikacin (0.210 μg/mL)			31.75	50.71±9.55
Rank	Top 3-Drug Combinations				IDentfi.AI %Inhibition	Measured %Inhibition
71	Levofloxacin (0.930 μg/mL)	Meropenem (2.996 μg/mL)	Rifabutin (0.0375 μg/mL)		48.01	58.33±4.99
97	Levofloxacin (0.930 μg/mL)	Meropenem (2.996 μg/mL)	Linezolid (0.966 μg/mL)		46.35	51.97±7.03
105	Levofloxacin (0.930 μg/mL)	Meropenem (2.996 μg/mL)	Clarithromycin (0.0442 μg/mL)		46.00	/
120	Levofloxacin (0.930 μg/mL)	Meropenem (2.996 μg/mL)	Amikacin (0.105 μg/mL)		45.35	/
129	Levofloxacin (0.930 μg/mL)	Meropenem (2.996 μg/mL)	Rifabutin (0.0188 μg/mL))		44.93	/
Rank	Top 4-Drug Combinations				IDentfi.AI %Inhibition	Measured %Inhibition
18	Levofloxacin (0.930 μg/mL)	Meropenem (2.996 μg/mL)	Amikacin (0.105 μg/mL)	Clarithromycin (0.0442 μg/mL)	52.56	51.75±6.01
19	Levofloxacin (0.930 μg/mL)	Meropenem (2.996 μg/mL)	Linezolid (0.966 μg/mL)	Rifabutin (0.0375 μg/mL)	52.52	55.80±6.56
26	Levofloxacin (0.930 μg/mL)	Meropenem (2.996 μg/mL)	Amikacin (0.105 μg/mL)	Rifabutin (0.0375 μg/mL)	51.52	/
36	Levofloxacin (0.930 μg/mL)	Meropenem (2.996 μg/mL)	Linezolid (0.966 μg/mL)	Clarithromycin (0.0442 μg/mL)	50.50	/
39	Levofloxacin (0.930 μg/mL)	Meropenem (2.996 μg/mL)	Linezolid (0.508 μg/mL)	Rifabutin (0.0375 μg/mL)	50.26	/

## Abbreviations

%Inhibition: percent inhibition
AI: artificial intelligence
AMK: amikacin
CDSS: clinical decision support system
CLR: clarithromycin
C_max_: maximum serum concentration of a drug achieved in the body
COPD: cystic fibrosis and chronic obstructive pulmonary disease
DiaMOND: diagonal measurement of n-way Drug Interactions
ESKAPE: Enterococcus faecium, Staphylococcus aureus, Klebsiella pneumoniae, Acinetobacter baumannii, Pseudomonas aeruginosa, and Enterobacter spp
FDA: food and drug administration
IC_10_: inhibitory concentration required to inhibit bacterial growth by 10 percent
IC_20_: inhibitory concentration required to inhibit bacterial growth by 20 percent
IC_50_: inhibitory concentration required to inhibit bacterial growth by 50 percent
IC_90_: inhibitory concentration required to inhibit bacterial growth by 90 percent
IV: intravenous
L0: level 0 concentration
L1: level 1 concentration
L2: level 2 concentration
Level 1 Conc.: level 1 concentration
Level 2 Conc.: level 2 concentration
LVX: levofloxacin
LZD: linezolid
*M. abscessus*: *Mycobacterium abscessus*
MEM: meropenem
MIC: minimum inhibitory concentration
MoA: mechanism of action
MTB: *mycobacterium tuberculosis*
NTM: nontuberculous mycobacteria
OACD: orthogonal array composite design
OD_600_: optimal density at a wavelength of 600 nm
RFB: rifabutin
SD: propagated standard deviation
SOC: standard of care
Z': z prime factor
