# Supplementary material for: Addressing antimicrobial resistance with the IDentif.AI platform: Rapidly optimizing clinically actionable combination therapy regimens against nontuberculous mycobacteria
Source: Theranostics. 2022 Sep 25;12(16):6848–64. doi: 10.7150/thno.73078 (PMC9576615; doi:10.7150/thno.73078)
Supplement: Supplementary file 1 — Supplementary figures and tables, information. [file thnov12p6848s1.pdf]

Addressing antimicrobial resistance with the IDentif.AI platform: Rapidly optimizing clinically actionable combination therapy regimens against nontuberculous mycobacteria

Devika Mukherjee, Peter Wang, Lissa Hooi, Vedant Sandhu, Kui You, Agata Blasiak*, Edward Kai-Hua Chow*, Dean Ho*, and Pui-Lai Rachel Ee*

*Corresponding Authors: agata.blasiak@nus.edu.sg (A.B.); csikce@nus.edu.sg (E.K.-H.C.); biedh@nus.edu.sg (D.H.); phaeplr@nus.edu.sg (P.L.R.E.)

This Supplementary Material includes:

Supplementary Text
Equations S1 to S3
Figures S1 to S4
Tables S1 to S9
Example Code
References

SUPPLEMENTART TEXT

Materials and Methods

Drugs

Linezolid (LZD; Sigma-Aldrich PZ0014), Clarithromycin (CLR; Sigma-Aldrich C9742), and Rifabutin (RFB; Sigma-Aldrich R3530) were dissolved in DMSO. Amikacin (AMK; Sigma-Aldrich 1019508), Meropenem (MEM; Sigma-Aldrich 1392454), and Levofloxacin (LVX; Sigma-Aldrich 28266) were dissolved in sterile-filtered water.

Drug Treatments in each Experiment

In the dose-response experiment, each drug was prepared by two-fold serial dilution. LZD, AMK, and MEM were tested in the range of 0.0977 µg/mL to 50 µg/mL (N = 2). CLR, RFB, and LVX were tested in ranges from 0.0244 µg/mL to 12.50 µg/mL, 0.0122 µg/mL to 6.25 µg/mL, and 0.00293 µg/mL to 3 µg/mL, respectively (N = 2).

The IDentif.AI combinatorial study was designed using a set of Orthogonal Array Composite Design (OACD)-designed drug combinations consisting of three clinically-relevant and actionable drug concentration levels (level 0, level 1, and level 2). The OACD design is based on a resolution VI 32-run fractional factorial (2-level; -1 and 1) and an 18-run orthogonal array (3-level; -1, 0, 1) as specified by Xu *et al* [1]. The three concentration levels according to OACD are level 0 (-1 in OACD), which indicates that a certain drug is absent in the combination, and level 1 and level 2 concentration levels (0 and 1 in OACD), which represent two clinically actionable concentrations selected using the dose response curves (Figure S1) or C_{max} values. The 50 OACD-designed combinations represent the minimum combinations needed to assess each drug's effects individually and in combinations through their linear, bilinear (drug-drug interactions), and quadratic parameters (coefficients) arising from the IDentif.AI regression analysis (N = 3) (Table S1). Additionally, the monotherapy efficacies for each of the drugs at level 1 and level 2 concentrations were also experimentally validated (N = 3) (Table 2).

The SOC, IDentif.AI-designed, and IDentif.AI-pinpointed non-effective combinations were validated *in vitro* (N = 3), and the concentrations were corresponded to the clinically actionable drug concentration levels in Table 2. Bliss independence model was performed to assess the drug interaction in LVX/RFB with concentration ranges: 0.00182 – 1.86 µg/mL for LVX and 0.00117 – 0.0750 µg/mL for RFB (N = 3) in a checkerboard assay. Furthermore, a DiaMOND interaction analysis for LVX/MEM was performed with concentration ranges: 0.116 – 3.720 µg/mL for LVX and 0.375 µg/mL – 11.984 µg/mL for MEM (N = 3).

Drug %Inhibition against *M. abscessus* was calculated using Equation S1.

$$\%Inhibiton = \left(1 - \frac{Drug\ Treatment}{Negative\ Control}\right) \times 100\% \quad \textbf{(S1)}$$

The Optical Density (OD_{600}) of combinations or monotherapies divided by the OD_{600} of negative controls (drug free) is equivalent to the viability of the bacteria. Using the equation above, %Inhibition against *M. abscessus* can then be determined for each monotherapy or combination.

Z'-factor for Assay Quality

To assess the quality of the MIC assay, Z Prime (Z')-factor was calculated according to Equation S2.

$$Z' = 1 - \frac{3(\sigma_{C-} + \sigma_{C+})}{|\mu_{c-} - \mu_{c+}|} \quad \textbf{(S2)}$$

In Equation S2, σ_{c+} and σ_{c} represent the standard deviation of positive control (media only) and negative control (drug free), respectively. μ_{c-} and μ_{c+} are the average of negative and positive controls, respectively [2].

Propagated Standard Deviations (SD)

$$\sigma_I^2 = \left(\frac{\partial I}{\partial E}\right)^2 \sigma_E^2 + \left(\frac{\partial I}{\partial c_-}\right)^2 \sigma_{c_-}^2 \quad \textbf{(S3)}$$

In Equation S3, σ_I is the propagated SD for the mean value of experimentally measured %Inhibitions. The propagated SD accounts for the spread of the raw OD_{600} values of experimental replicates and negative control (drug free), which are represented by σ_E and σ_{c-}, respectively [3].

Statistical Analysis

In this study, the sample distribution of IDentif.AI-designed combinations was tested using the Shapiro-Wilk normality test. For multiple comparison and pairwise comparison, Kruskal-Wallis test and Dunn's post hoc test were performed, respectively.

Results

C_{max} Selection

One of the parameters for dose selection was based on C_{max} of the drugs. To determine the C_{max} values, Food and Drug Administration (FDA) regulatory documents and literatures were referenced. Following a single oral dose administration of 600 mg LZD, the C_{max} reached 12.70 µg/mL [4]. 590 mg of Amikacin administered via oral inhalation in NTM adult patients resulted in a C_{max} of 2.10 µg/mL [5]. In a cohort of healthy volunteers, the steady-state C_{max} of MEM was 61.60 µg/mL via intravenous infusion [6]. In an FDA regulatory document, CLR had a reported stead-state C_{max} of 10 µg/mL at 1000 mg twice daily (bid) [7]. In 9 healthy volunteers, RFB had a reported C_{max} of 0.375 µg/mL following a single oral dose of 300 mg [8]. A single oral dose administration of LVX at 750 mg resulted in a steady-state C_{max} of 9.30 µg/mL [9].

Assay Quality for each Experiment

The assay quality for the dose-response experiment was deemed "excellent" as indicated by a Z'-factor of 0.723 (N = 42). In the subsequent IDentif.AI analysis, the assay quality was also considered "excellent" with a Z'-factor of 0.828 (N = 33). The validation study including synergy analyses had an "excellent" assay with a Z'-factor of 0.589 (N = 15).

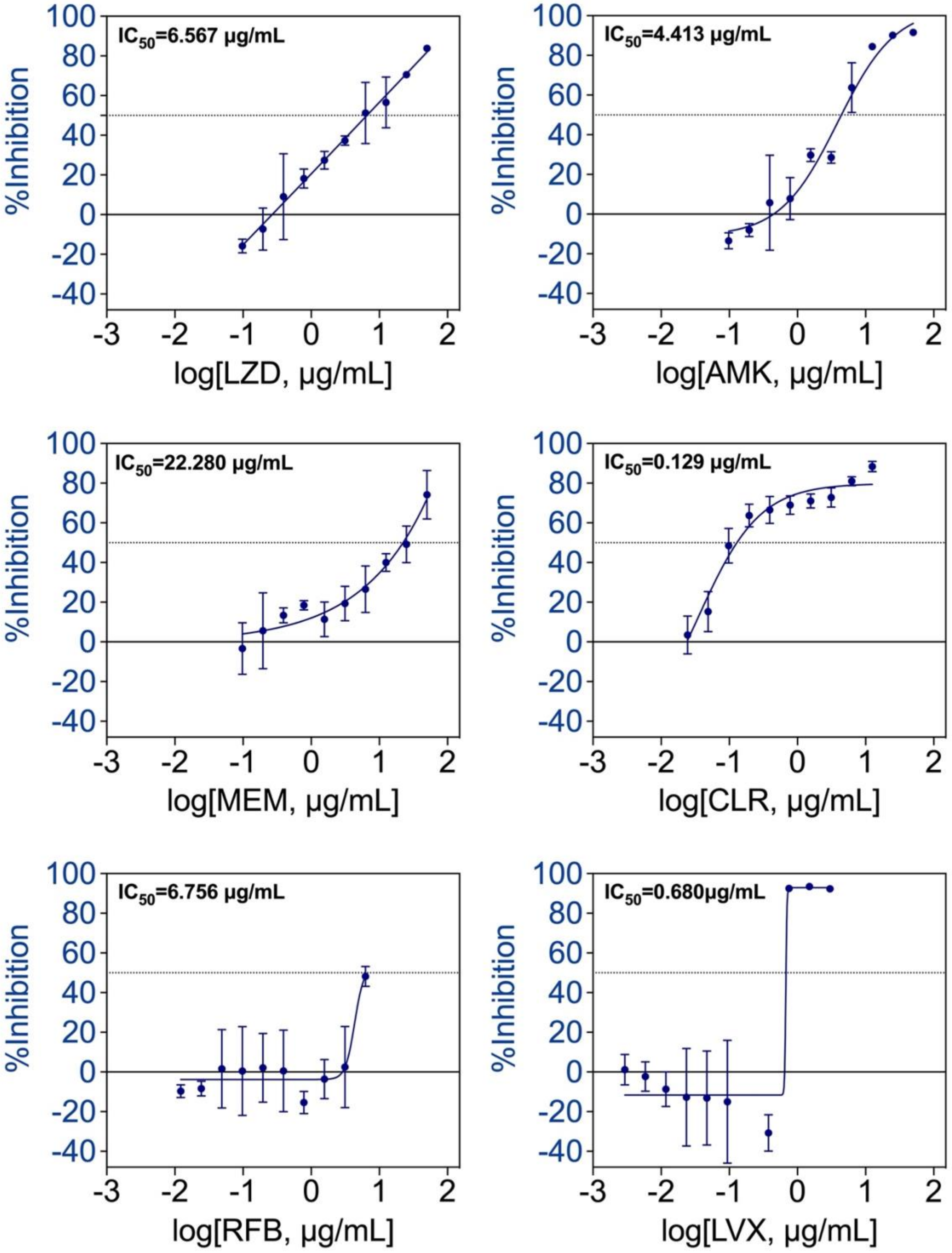

Figure S1. Dose response curves for all 6 antibiotics. *M. abscessus subsp abscessus* was grown in Middlebrook 7H9 broth (BD) supplemented with ADC (Sigma Aldrich). Cultures were grown in mid-log phase and then diluted to a final density of 10^6 CFU/mL in the drug containing microtiter plates. The bacterial growth %Inhibition resulted from the drugs in monotherapies was

determined by measuring the OD_{600} after 72 h of incubation at 37°C with shaking at 120 rpm. The dotted line represents absolute IC_{50}. Data points are presented as mean ± propagated SD (N = 2). AMK: amikacin, CLR: clarithromycin, LVX: levofloxacin, LZD: linezolid, MEM: meropenem, and RFB: rifabutin.

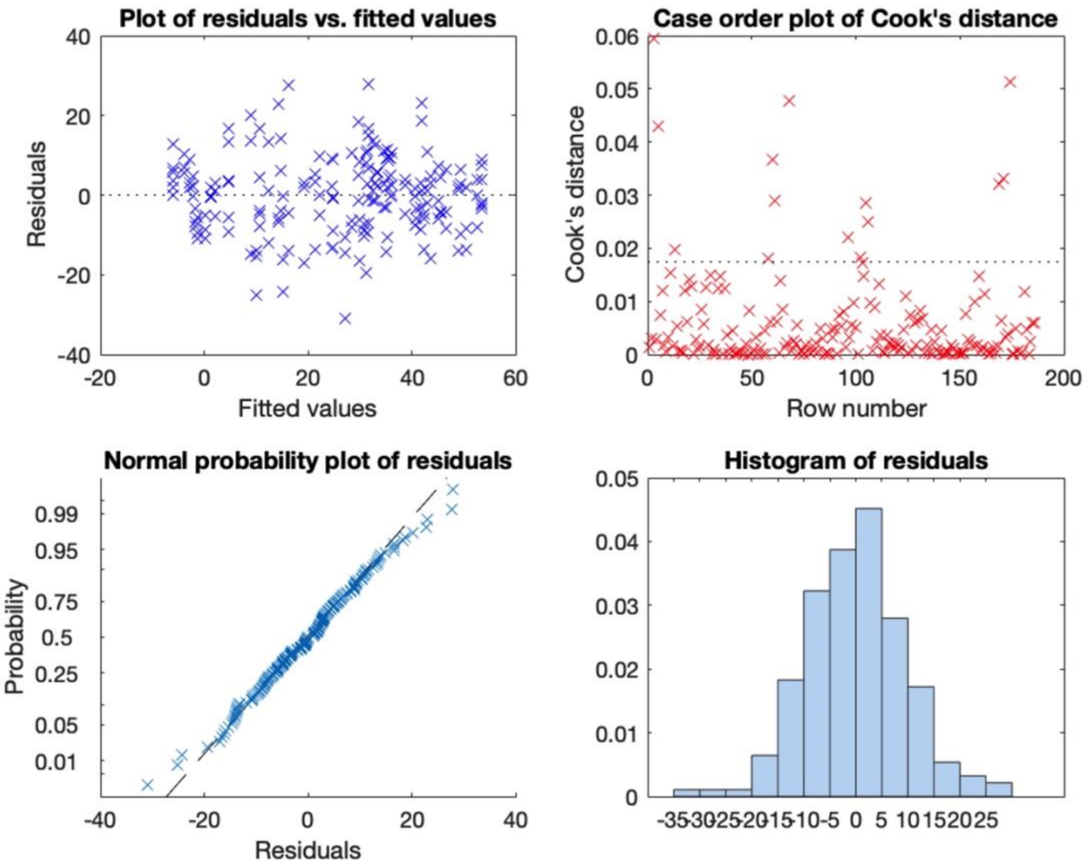

Figure S2. IDentif.AI residual-based outlier analysis for the %Inhibition data. In the IDentif.AI outlier analysis, all OACD-designed combinations (N = 3) and drugs in monotherapies (level 1 and level 2) (N = 3) were used in the IDentif.AI regression analysis. In this analysis, residual is defined as the difference between IDentif.AI-predicted %Inhibition and the experimentally measured %Inhibition. The plot of residuals vs. fitted values assessed the distribution of residuals for all OACD-designed combinations including the monotherapies, and this plot also examines the suitability of IDentif.AI quadratic model fit. In the Cook's distance plot, the row numbers represent each OACD-designed combinations and monotherapies in order (Table S1). The normality of residual distribution was assessed through the normal probability plot and the histogram of residuals. This series of residual-based outlier analyses did not identify any outlier, and no data was removed for the IDentif.AI analysis.

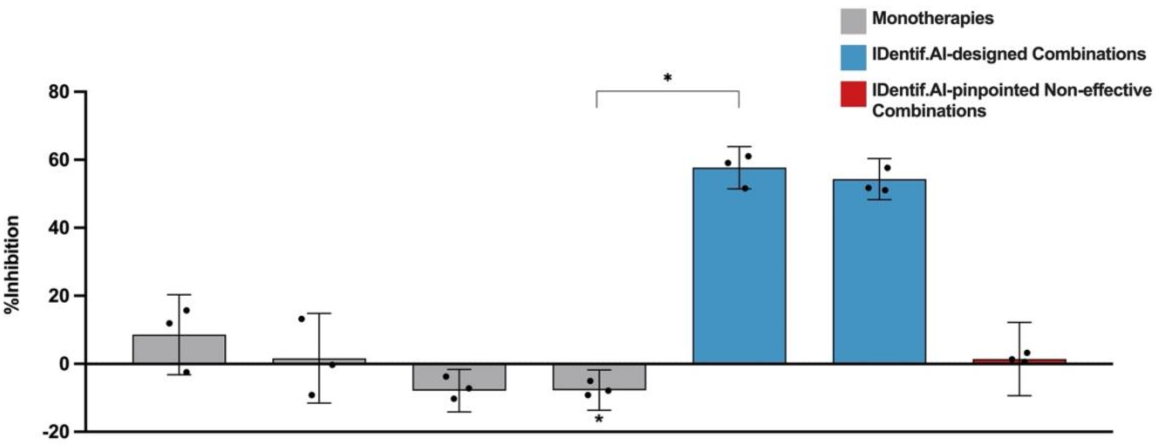

LVX (μg/mL)	0.930	0	0	0	0.930	0.930	0
MEM (μg/mL)	0	2.996	0	0	2.996	0	0
CLR (μg/mL)	0	0	0	0.0442	0	0	0.0442
RFB (μg/mL)	0	0	0.0375	0	0	0.0375	0.0375

Figure S3. Validation of IDentif.AI-designed 2-drug combinations and monotherapies. IDentif.AI-designed 2-drug combinations (blue) were compared to their respective monotherapies (gray). The non-effective combinations (RFB/CLR) (red) pinpointed by IDentif.AI were also compared to monotherapies and LVX/RFB (blue). The concentrations with respect to each monotherapy and combination are listed in the table below. Data points are presented as mean ± propagated SD. Error bars represent the propagated SD (N = 3). Kruskal-Wallis test detected statistically significant differences at $P < 0.05$ for the %Inhibitions among monotherapies and combinations. Subsequently, pairwise comparisons via Dunn's post hoc test identified statistically significant differences between CLR in monotherapy and LVX/MEM (*$P < 0.05$). CLR: clarithromycin, LVX: levofloxacin, MEM: meropenem, and RFB: rifabutin.

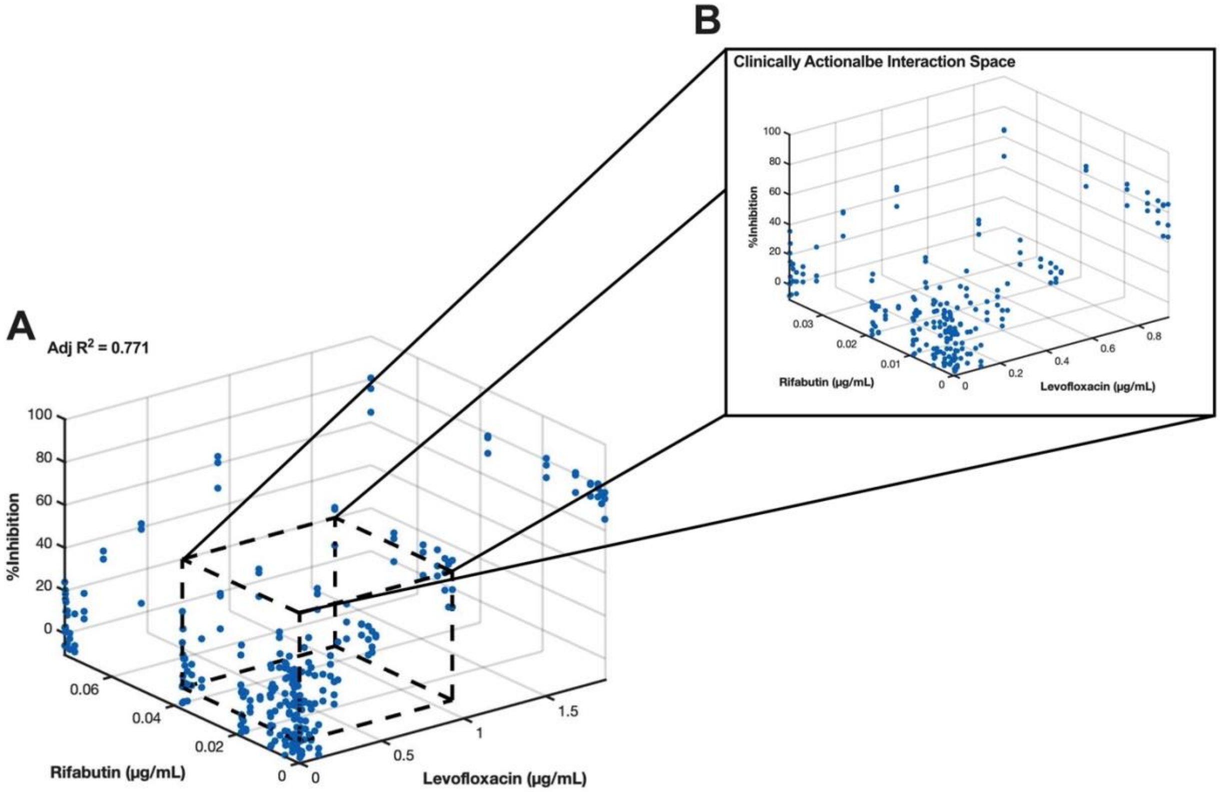

Figure S4. Individual replicates of the checkerboard assay data used to construct LVX/RFB response surfaces. (A) Individual replicates (N = 3) of LVX/RFB in the validation interaction space (0% - 20% C_{max}) and **(B)** the clinically actionable interaction space (< 10% C_{max}) are in blue dots. The black dotted box in Figure S4A represents the clinically actionable interaction space. LVX: levofloxacin and RFB: rifabutin.

Table S1. Resolution VI 6-drug OACD design. 50 OACD-designed combinations at three different concentration levels. Level 0, or -1 in the table, indicates that a given drug is not present in the combination [1]. Level 1 and level 2, or 0 and 1 in the table respectively, represent two concentration levels selected using parameters obtained from dose response curves or C_{max} values. AMK: amikacin, CLR: clarithromycin, LVX: levofloxacin, LZD: linezolid, MEM: meropenem, and RFB: rifabutin.

Combination	LZD	AMK	MEM	CLR	RFB	LVX
1	-1	-1	-1	-1	-1	-1
2	1	-1	-1	-1	-1	1
3	-1	1	-1	-1	-1	1
4	1	1	-1	-1	-1	-1
5	-1	-1	1	-1	-1	1
6	1	-1	1	-1	-1	-1
7	-1	1	1	-1	-1	-1
8	1	1	1	-1	-1	1
9	-1	-1	-1	1	-1	1
10	1	-1	-1	1	-1	-1
11	-1	1	-1	1	-1	-1
12	1	1	-1	1	-1	1
13	-1	-1	1	1	-1	-1
14	1	-1	1	1	-1	1
15	-1	1	1	1	-1	1
16	1	1	1	1	-1	-1
17	-1	-1	-1	-1	1	1
18	1	-1	-1	-1	1	-1
19	-1	1	-1	-1	1	-1
20	1	1	-1	-1	1	1
21	-1	-1	1	-1	1	-1
22	1	-1	1	-1	1	1
23	-1	1	1	-1	1	1
24	1	1	1	-1	1	-1
25	-1	-1	-1	1	1	-1
26	1	-1	-1	1	1	1
27	-1	1	-1	1	1	1
28	1	1	-1	1	1	-1
29	-1	-1	1	1	1	1

30	1	-1	1	1	1	-1
31	-1	1	1	1	1	-1
32	1	1	1	1	1	1
33	-1	-1	-1	-1	-1	-1
34	-1	0	0	0	0	0
35	-1	1	1	1	1	1
36	0	-1	-1	0	0	1
37	0	0	0	1	1	-1
38	0	1	1	-1	-1	0
39	1	-1	0	-1	1	0
40	1	0	1	0	-1	1
41	1	1	-1	1	0	-1
42	-1	-1	1	1	0	0
43	-1	0	-1	-1	1	1
44	-1	1	0	0	-1	-1
45	0	-1	0	1	-1	1
46	0	0	1	-1	0	-1
47	0	1	-1	0	1	0
48	1	-1	1	0	1	-1
49	1	0	-1	1	-1	0
50	1	1	0	-1	0	1

Table S2. IDentif.AI estimated coefficients for %Inhibition data analysis. AMK: amikacin, CLR: clarithromycin, LVX: levofloxacin, LZD: linezolid, MEM: meropenem, and RFB: rifabutin. Statistical significance was determined using *F*-test. *$P < 0.05$, ** $P < 0.01$, and *** $P < 0.001$.

	**Estimate**	**Statistical Significance**
Intercept	35.136	***
LZD	5.1383	***
AMK	1.9575	*
MEM	7.0936	***
CLR	1.9786	*
RFB	1.4563	
LVX	12.06	***
LZD:LVX	-2.8852	***
AMK:MEM	-1.8293	*
AMK:CLR	1.5325	
MEM:LVX	-1.6231	
CLR:RFB	-1.6289	
AMK^2	-4.914	*
Degrees of Freedom	173	
Correlation Coefficient	0.872	
Adj. R^2 (IDentif.AI)	0.744	
R^2 (IDentif.AI)	0.761	
F-test	***	

Table S3. Monotherapy results for all 6 drugs at level 1 and level 2 concentrations. The experimentally measured %Inhibitions of all 6 drugs at level 1 and level 2 concentrations (N = 3). AMK: amikacin, CLR: clarithromycin, LVX: levofloxacin, LZD: linezolid, MEM: meropenem, and RFB: rifabutin.

**%Inhibition for Level 1 Conc.**					
**Drug**	**Replicate 1**	**Replicate 2**	**Replicate 3**	**Average**	**Propagated SD**
LZD	5.11	-3.22	3.26	1.72	6.67
AMK	3.13	0.66	0.88	1.56	5.22
MEM	21.25	8.28	17.92	15.82	8.00
CLR	6.62	3.23	1.64	3.83	5.54
RFB	6.15	0.14	-0.79	1.83	6.28
LVX	2.94	2.80	5.90	3.88	5.22
**%Inhibition for Level 2 Conc.**					
**Drug**	**Replicate 1**	**Replicate 2**	**Replicate 3**	**Average**	**Propagated SD**
LZD	-3.80	-15.02	-5.15	-7.99	8.25
AMK	-7.00	-12.19	-10.73	-9.97	6.23
MEM	-0.98	-9.07	14.89	1.62	13.19
CLR	-5.17	-9.44	-8.56	-7.72	5.96
RFB	-4.75	-8.41	-10.46	-7.87	6.23
LVX	12.73	-3.67	16.62	8.56	11.74

Table S4. %Inhibition data for all 50 OACD-designed combinations. The order of the combinations is in accordance to Table S1 (N = 3).

	%Inhibition				
**Combination**	**Replicate 1**	**Replicate 2**	**Replicate 3**	**Average**	**Propagated SD**
1	0.69	-0.06	-3.89	-1.09	7.65
2	38.52	24.73	48.36	37.20	10.99
3	59.56	31.02	46.71	45.77	13.80
4	20.92	10.74	28.75	20.14	8.61
5	64.90	42.97	60.61	56.16	10.98
6	41.86	39.41	11.78	31.02	18.94
7	25.73	13.47	0.44	13.21	15.11
8	48.40	27.71	46.71	40.94	10.78
9	34.13	21.97	42.59	32.89	9.63
10	7.53	37.03	8.44	17.67	19.29
11	-5.75	29.05	22.59	15.30	19.73
12	42.27	38.26	52.83	44.45	7.18
13	2.37	21.28	22.33	15.32	12.54
14	42.02	36.79	52.46	43.75	7.55
15	52.01	39.55	55.81	49.12	7.90
16	41.32	42.61	33.99	39.31	7.18
17	35.63	39.36	39.04	38.01	4.71
18	2.45	43.99	11.92	19.45	23.84
19	-4.53	8.17	-0.72	0.97	11.20
20	28.82	36.52	37.61	34.31	6.41
21	7.74	26.59	24.75	19.69	11.87
22	52.45	44.44	56.43	51.11	5.78
23	39.42	43.87	52.54	45.28	6.92
24	31.41	37.37	45.77	38.18	7.63
25	2.62	-2.32	0.07	0.12	5.77
26	44.91	39.94	44.32	43.06	3.97
27	33.14	44.70	38.69	38.84	7.66
28	11.18	26.73	33.83	23.91	12.22
29	41.16	41.34	50.06	44.19	5.37
30	20.87	31.87	44.30	32.35	11.87
31	22.34	17.42	32.05	23.94	7.40
32	50.06	50.85	62.41	54.44	6.75
33	-5.88	6.94	-2.18	-0.37	8.74
34	13.50	38.24	23.77	25.17	14.30
35	35.05	50.31	51.13	45.50	9.74
36	31.76	36.08	45.46	37.76	7.34
37	11.77	24.10	24.36	20.08	8.97
38	21.95	20.67	23.66	22.10	4.90
39	35.73	45.23	41.12	40.69	6.65
40	57.26	61.04	51.41	56.57	6.51
41	15.89	24.29	33.70	24.63	9.47

42	31.10	46.12	33.59	36.94	10.05
43	42.27	32.92	40.37	38.52	5.44
44	6.63	27.32	15.12	16.36	12.81
45	37.58	35.12	37.63	36.78	4.03
46	28.44	48.16	34.92	37.17	11.72
47	20.16	38.84	22.06	27.02	12.59
48	36.23	46.94	38.72	40.63	7.66
49	24.72	46.89	37.21	36.27	12.46
50	44.02	53.97	40.97	46.32	8.70

Table S5. Experimental data for the validated combinations. The combinations tabulated here are in accordance to Figure 4 (N = 3). AMK: amikacin, CLR: clarithromycin, LVX: levofloxacin, LZD: linezolid, MEM: meropenem, and RFB: rifabutin. Drug 1 (D1), Drug 2 (D2), Drug 3 (D3), and Drug 4 (D4). Replicate 1 (Rep. 1), Replicate 2 (Rep. 2), and Replicate 3 (Rep. 3).

				%Inhibition				
**D1**	**D2**	**D3**	**D4**	**Rep. 1**	**Rep. 2**	**Rep. 3**	**Average**	**Propagated SD**
**Standard of Care Combinations**								
AMK	CLR	RFB		21.41	42.51	33.61	32.51	12.87
AMK	CLR	LZD		47.67	37.73	45.54	43.65	8.04
**IDentif.AI-designed Combinations**								
LVX	MEM			59.01	53.00	61.07	57.69	6.21
LVX	RFB			51.85	52.90	58.21	54.32	6.01
LVX	AMK			45.03	47.34	59.76	50.71	9.55
LVX	MEM	RFB		57.43	56.80	60.76	58.33	4.99
LVX	MEM	LZD		57.37	48.58	49.97	51.97	7.03
LVX	MEM	AMK	CLR	52.48	54.28	48.49	51.75	6.01
LVX	MEM	LZD	RFB	60.24	51.27	55.89	55.80	6.56
**IDentif.AI-pinpointed Non-effective Combinations**								
MEM	AMK	RFB		23.17	33.93	23.70	26.93	9.97
CLR	LZD	RFB		17.19	11.83	13.37	14.13	9.70
CLR	RFB			1.26	3.09	-0.17	1.39	10.80

Table S6. IDentif.AI-designed combinations and non-effective combinations. The rank (out of 729 total combinations) and IDentif.AI-predicted and measured %Inhibitions for each combination are listed in the table. The concentration levels are summarized in the parenthesis.

**Rank**	**Drug 1**	**Drug 2**	**Drug 3**	**Drug 4**	**Measured %Inhibition**	**Predicted %Inhibition**
190	LVX (L2)	MEM (L2)	/	/	57.69±6.21	41.84
363	LVX (L2)	RFB (L2)	/	/	54.32±6.01	33.41
396	LVX (L2)	AMK (L2)	/	/	50.71±9.55	31.75
71	LVX (L2)	MEM (L2)	RFB (L2)	/	58.33±4.99	48.01
97	LVX (L2)	MEM (L2)	LZD (L2)	/	51.97±7.03	46.35
18	LVX (L2)	MEM (L2)	AMK (L1)	CLR (L2)	51.75±6.01	52.56
19	LVX (L2)	MEM (L2)	LZD (L2)	RFB (L2)	55.80±6.56	52.52
688	AMK (L2)	MEM (L1)	RFB (L1)	/	26.93±9.97	8.59
705	CLR (L2)	RFB (L1)	LZD (L1)	/	14.13±9.70	6.11
725	CLR (L2)	RFB (L2)	/	/	1.39±10.80	-2.09

Table S7. Experimental data for LVX/RFB checkerboard assay. All replicates of the checkerboard assay are tabulated (N = 3). LVX: levofloxacin and RFB: rifabutin. Replicate 1 (Rep. 1), Replicate 2 (Rep. 2), and Replicate 3 (Rep. 3).

Concentration (μg/mL)		%Inhibition				
**LVX**	**RFB**	**Rep. 1**	**Rep. 2**	**Rep. 3**	**Average**	**Propagated SD**
0	0	0	0	0	0	0
1.860	0	65.17	74.67	77.65	72.49	4.57
0.930	0	43.50	51.54	65.29	53.44	8.97
0.465	0	39.15	39.97	39.65	39.59	6.99
0.233	0	41.60	-14.82	23.89	16.89	36.91
0.116	0	-5.51	5.37	-8.47	-2.87	13.93
0.0581	0	16.56	2.00	-4.07	4.83	22.58
0.0291	0	-27.99	-12.45	0.40	-13.35	12.28
0.0145	0	-18.30	5.92	-4.98	-5.79	12.25
0.00727	0	-6.64	6.29	-4.60	-1.65	13.36
0.00363	0	21.48	17.27	-7.50	10.41	24.22
0.00182	0	19.01	-5.43	3.87	5.82	23.41
0	0.0750	2.78	24.04	-5.47	7.12	17.97
0	0.0375	-7.27	21.01	0.64	4.80	12.49
0	0.0188	13.92	27.92	-5.03	12.27	21.48
0	0.00938	7.09	15.58	-2.25	6.81	15.17
0	0.00469	-2.56	31.28	-9.50	6.41	22.08
0	0.00234	1.60	27.64	-4.50	8.25	18.59
0	0.00117	-31.82	20.06	0.95	-3.60	31.64
1.860	0.00117	71.66	76.36	76.80	74.94	2.19
0.930	0.00117	42.58	62.85	63.52	56.32	8.35
0.465	0.00117	31.22	41.07	33.84	35.38	7.13
0.233	0.00117	16.34	22.17	11.81	16.77	11.39
0.116	0.00117	27.55	14.69	1.64	14.63	21.65
0.0581	0.00117	-25.82	7.76	3.98	-4.69	16.01
0.0291	0.00117	16.28	8.44	-1.19	7.84	18.66
0.0145	0.00117	24.87	14.85	-1.16	12.85	21.90
0.00727	0.00117	28.93	5.88	-3.21	10.54	26.92
0.00363	0.00117	18.28	20.50	-4.32	11.49	21.02
0.00182	0.00117	17.40	30.72	-10.99	12.38	27.04
1.860	0.00234	74.05	77.93	80.12	77.37	2.31
0.930	0.00234	49.84	57.90	64.54	57.43	5.48
0.465	0.00234	36.35	42.40	29.13	35.96	10.81
0.233	0.00234	33.86	29.51	24.12	29.16	12.11
0.116	0.00234	30.49	11.27	-3.49	12.76	26.54
0.0581	0.00234	29.74	11.97	-8.77	10.98	29.37
0.0291	0.00234	24.92	15.56	10.25	16.91	16.09
0.0145	0.00234	23.56	10.09	8.70	14.12	17.35
0.00727	0.00234	16.37	15.27	-5.32	8.77	20.60
0.00363	0.00234	20.74	29.36	-2.20	15.97	21.98

0.00182	0.00234	25.44	30.29	-6.62	16.37	26.51
1.860	0.00469	72.67	78.26	78.83	76.58	2.33
0.930	0.00469	54.83	59.57	66.97	60.46	5.09
0.465	0.00469	42.17	41.82	36.88	40.29	8.86
0.233	0.00469	23.32	11.01	15.96	16.76	14.72
0.116	0.00469	38.25	13.44	12.83	21.51	22.37
0.0581	0.00469	29.33	6.16	-0.84	11.55	25.74
0.0291	0.00469	29.79	9.05	-1.60	12.41	25.59
0.0145	0.00469	26.37	-1.78	1.36	8.65	26.27
0.00727	0.00469	32.77	-4.66	-10.32	5.93	40.32
0.00363	0.00469	26.06	15.53	-5.07	12.17	24.76
0.00182	0.00469	19.08	33.83	1.64	18.18	20.36
1.860	0.00938	71.32	79.39	80.53	77.08	3.42
0.930	0.00938	52.03	62.76	66.17	60.32	5.03
0.465	0.00938	39.87	48.32	32.04	40.08	11.37
0.233	0.00938	37.42	25.68	20.72	27.94	15.77
0.116	0.00938	31.14	4.85	8.26	14.75	23.23
0.0581	0.00938	33.45	9.47	-4.58	12.78	29.11
0.0291	0.00938	18.15	7.28	-6.68	6.25	23.28
0.0145	0.00938	20.23	16.88	0.82	12.64	18.55
0.00727	0.00938	27.79	18.61	-8.24	12.72	27.46
0.00363	0.00938	5.30	14.19	-8.76	3.58	18.52
0.00182	0.00938	0.54	29.38	1.13	10.35	16.42
1.860	0.0188	72.08	77.92	80.94	76.98	3.19
0.930	0.0188	51.99	62.89	65.34	60.08	4.78
0.465	0.0188	39.46	46.54	49.21	45.07	4.73
0.233	0.0188	33.63	31.24	17.07	27.31	15.34
0.116	0.0188	6.00	4.73	5.67	5.47	11.68
0.0581	0.0188	2.31	5.49	-0.02	2.59	12.00
0.0291	0.0188	-7.36	9.72	-8.18	-1.94	15.68
0.0145	0.0188	7.98	10.71	-3.16	5.18	15.78
0.00727	0.0188	-14.84	11.09	-1.58	-1.77	17.42
0.00363	0.0188	-13.71	32.30	-0.14	6.15	18.26
0.00182	0.0188	10.28	10.73	-8.22	4.26	20.23
1.860	0.0375	70.68	78.00	78.83	75.84	3.00
0.930	0.0375	47.09	63.97	64.83	58.63	6.87
0.465	0.0375	32.78	45.76	43.85	40.80	5.35
0.233	0.0375	39.13	38.06	22.68	33.29	14.77
0.116	0.0375	20.24	1.08	-2.34	6.33	23.67
0.0581	0.0375	4.69	-0.36	9.63	4.66	12.29
0.0291	0.0375	0.90	7.17	-7.01	0.35	17.41
0.0145	0.0375	10.06	13.30	1.85	8.40	13.84
0.00727	0.0375	-13.88	13.36	-6.65	-2.39	17.97
0.00363	0.0375	15.22	27.43	5.17	15.94	15.56
0.00182	0.0375	21.04	35.49	0.39	18.97	22.08
1.860	0.0750	64.73	75.77	80.72	73.74	6.13

0.930	0.0750	48.55	60.57	63.45	57.52	5.33
0.465	0.0750	39.03	41.62	4.55	28.40	26.13
0.233	0.0750	29.84	29.81	33.66	31.10	7.69
0.116	0.0750	16.32	4.54	8.09	9.65	15.78
0.0581	0.0750	8.10	-9.74	-6.84	-2.83	16.65
0.0291	0.0750	-3.16	-0.48	-8.10	-3.91	13.52
0.0145	0.0750	10.32	8.27	-4.99	4.53	18.21
0.00727	0.0750	14.64	10.14	-14.12	3.56	28.00
0.00363	0.0750	18.34	20.26	3.16	13.92	16.53
0.00182	0.0750	2.10	16.21	0.80	6.37	12.18

Table S8. Model statistics for LVX/RFB response surface. The statistics for LVX/RFB response surface are tabulated below. Statistical significance was determined using *F*-test. ***$P <$ 0.001. LVX: levofloxacin and RFB: rifabutin.

**Statistics**	**LVX/RFB**
Observations	288
Degrees of Freedom	282
Correlation Coefficient	0.881
Adj R^2	0.771
R^2	0.775
F-test	***

Table S9. Experimental data for LVX/MEM DiaMOND synergy analysis. All replicates of the synergy analysis are tabulated (N = 3). LVX: levofloxacin and MEM: meropenem. Replicate 1 (Rep. 1), Replicate 2 (Rep. 2), and Replicate 3 (Rep. 3).

Concentration (μg/mL)		%Inhibition				
**LVX**	**MEM**	**Rep. 1**	**Rep. 2**	**Rep. 3**	**Average**	**Propagated SD**
0	0	0	0	0	0	0
3.720	0	82.58	85.51	88.02	85.37	3.72
1.860	0	69.24	70.66	75.84	71.91	5.98
0.930	0	58.24	56.69	59.64	58.19	7.40
0.465	0	45.27	50.82	47.76	47.95	9.44
0.233	0	-23.48	-20.45	-16.41	-20.11	21.12
0.11625	0	16.81	-18.18	-11.88	-4.42	25.99
0	11.984	-20.01	20.13	9.84	3.32	26.75
0	5.992	-29.59	-19.62	-10.57	-19.93	22.86
0	2.996	5.69	-16.41	11.18	0.16	22.64
0	1.498	-8.89	-3.05	16.27	1.44	21.57
0	0.749	-14.78	-21.63	16.56	-6.62	27.50
0	0.375	-33.20	-17.16	-18.04	-22.80	23.11
3.720	11.984	83.05	85.03	85.29	84.46	2.96
1.860	5.992	75.20	72.13	78.93	75.42	5.45
0.930	2.996	60.47	62.85	73.95	65.76	9.33
0.465	1.498	24.33	47.86	16.55	29.58	20.36
0.233	0.749	28.51	30.84	29.07	29.47	12.28
0.11625	0.375	-40.97	-24.11	-20.51	-28.53	24.81

EXAMPLE CODE

```
%Load OACD Data and Respective %Inhibitions

data =
[
```

Linezolid	Amikacin	Meropenem	Clarithromycin	Rifabutin	Levofloxacin	%Inhibition
-1	-1	-1	-1	-1	-1	0.69
1	-1	-1	-1	-1	1	38.52
-1	1	-1	-1	-1	1	59.56
1	1	-1	-1	-1	-1	20.92
-1	-1	1	-1	-1	1	64.90
1	-1	1	-1	-1	-1	41.86
-1	1	1	-1	-1	-1	25.73
1	1	1	-1	-1	1	48.40
-1	-1	-1	1	-1	1	34.13
1	-1	-1	1	-1	-1	7.53
-1	1	-1	1	-1	-1	-5.75
1	1	-1	1	-1	1	42.27
-1	-1	1	1	-1	-1	2.37
1	-1	1	1	-1	1	42.02
-1	1	1	1	-1	1	52.01
1	1	1	1	-1	-1	41.32
-1	-1	-1	-1	1	1	35.63
1	-1	-1	-1	1	-1	2.45
-1	1	-1	-1	1	-1	-4.53
…	…	…	…	…	…	…

```
]

%Define Inputs and Outputs

x = data(: , 1:6);
y = data(: , 8);


%IDentif.AI Quadratic Series

result = stepwiselm(x , y, ‘quadratic’, 'ResponseVar', 'Inhibition', 'PredictorVars', {‘Linezolid’,
‘Amikacin’, ‘Meropenem’, ‘Clarithromycin’, ‘Rifabutin’, ‘Levofloxacin’});
```

REFERENCES

1. Xu H, Jaynes J, Ding X. Combining two-level and three-level orthogonal arrays for factor screening and response surface exploration. Stat Sin. 2014; 24: 269-89.
2. Zhang J-H, Chung TD, Oldenburg KR. A simple statistical parameter for use in evaluation and validation of high throughput screening assays. J Biomol Screen. 1999; 4: 67-73.
3. Farrance I, Frenkel R. Uncertainty of measurement: A review of the rules for calculating uncertainty components through functional relationships. Clin Biochem Rev. 2012; 33: 49.
4. [Internet] FDA. Linezolid: Highlights of prescribing information. 2013. https://www.accessdata.fda.gov/drugsatfda_docs/label/2014/021130s032,021131s026,021132s031lbl.pdf
5. [Internet] FDA. Amikacin liposome inhalation suspension (ALIS) meeting of the antimicrobial drugs advisory committee (AMDAC). 2018. https://www.fda.gov/media/114875/download
6. [Internet] Antimicrobe. Mean pharmacokinetics parameters of carbapenems in healthy volunteers at steady state after intravenous infusions. http://www.antimicrobe.org/d12tab.htm
7. [Internet] FDA. Clarithromycin: Highlights of prescribing information. 2017. https://www.accessdata.fda.gov/drugsatfda_docs/label/2017/050662s058,050698s038,050775s026lbl.pdf
8. [Internet] FDA. Mycobutin. 2008. https://www.accessdata.fda.gov/drugsatfda_docs/label/2008/050689s016lbl.pdf
9. [Internet] FDA. Levofloxacin: Highlgihts of prescribing information. 2007. https://www.accessdata.fda.gov/drugsatfda_docs/label/2018/020634s069lbl.pdf
